# Investigation of Drag Reduction Technologies for Light-Duty Vehicles Using Surface, Wake and Underbody Pressure Measurements to Complement Aerodynamic Drag Measurements

**DOI:** 10.4271/2019-01-0644

**Published:** 2019

**Authors:** Fenella de Souza, Arash Raeesi, Marc Belzile, Cheryl Caffrey, Andreas Schmitt

**Affiliations:** National Research Council Canada; National Research Council Canada; Transport Canada; US EPA; Röchling Automotive SE & Co. KG

**Keywords:** drag reduction, surface pressure, wake pressure

## Abstract

Amulti-year, multi-vehicle study was conducted to quantify the aerodynamic drag changes associated with drag reduction technologies for light-duty vehicles. Various technologies were evaluated through fullscale testing in a large low-blockage closed-circuit wind tunnel equipped with a rolling road, wheel rollers, boundary-layer suction and a system to generate road-representative turbulent winds. The technologies investigated include active grille shutters, production and custom underbody treatments, air dams, wheel curtains, ride height control, side mirror removal and combinations of these.

This paper focuses on mean surface-, wake-, and underbody-pressure measurements and their relation to aerodynamic drag. Surface pressures were measured at strategic locations on four sedans and two crossover SUVs. Wake total pressures were mapped using a rake of Pitot probes in two cross-flow planes at up to 0.4 vehicle lengths downstream of the same six vehicles in addition to a minivan and a pick-up truck. A smaller rake was used to map underbody total pressures in one cross-flow plane downstream of the rear axle for three of these vehicles.

The results link drag reduction due to various technologies with specific changes in vehicle surface, rear underbody and wake pressures, and provide a database for numerical studies. In particular, the results suggest that existing or idealized prototype technologies such as active grille shutters, sealing the external grille and ride height control reduce drag by redirecting incoming flow from the engine bay or underbody region to smoother surfaces above and around the vehicle. This mechanism can enhance the reduction in wheel drag due to reduced wheel exposure at lowered ride height. Sealing the external grille was found to redirect the flow more efficiently than closing the grille shutters, and resulted in greater drag reduction. Underbody treatments were also found in some cases to redistribute the flow around the vehicle to reduce pressure drag in addition to underbody friction drag. The magnitude and spatial extent of the measured pressure changes due to the various technologies were often consistent with the amount of drag reduction.

## Introduction

Vehicle aerodynamic performance is a highly technical and complex issue. Adding a feature such as an air dam or active grille shutter to a vehicle optimized aerodynamically in its absence may not lead to the expected improvement in performance. Rather, a manufacturer may need to design a new vehicle as an “integrated system”, particularly when seeking to maximize the benefits from newer adaptive aerodynamic features. Due to the proprietary nature of automotive design and development, limited public information exists about the optimal design of passenger vehicle aerodynamics, including how beneficial some of the newer aerodynamic features may be in the “real-world”. Furthermore, current U.S. and Canadian test procedures used to demonstrate compliance with regulated GHG standards may not fully reflect the GHG benefits of these features. Instead, the U.S. and Canadian regulations provide “allowances” or “credits” that aim to encourage and recognize innovative technologies such as adaptive aerodynamics. The multi-year study commissioned by Transport Canada [1, 2] may help to assess these allowances or credits for a variety of vehicle US EPA size classes. By providing a better understanding of how aerodynamic technologies affect the flow around specific vehicles, the surface-, underbody- and wake-pressure measurements described in this paper can provide insight into why certain technologies are more or less effective for different vehicles.

Measurements were carried out on a series of 2015 to 2017 model-year production vehicles. The objective was to relate the drag reduction performance of aerodynamic treatments to specific changes in the flow around a vehicle. Treatments are add-on parts or modifications which primarily serve an aerodynamic function as opposed to aerodynamic features designed into the shape of the body. The treatments investigated included active grille shutters and/or grille sealing, ride height changes, original equipment manufacturer (OEM) and/ or custom underbody covers, wheel curtains, air dams, side mirror removal and combinations of these.

The test vehicles were chosen to represent a range of “advanced” aerodynamics models. Many have an OEM *ECO* designation suggesting that the vehicle is marketed as having components or parts installed to improve fuel efficiency and reduce environmental impacts. Attention was given to choose vehicles from different utility and price segments. The vehicles were selected by the project steering committee composed of representatives from government regulators, government research organizations and technology suppliers. Out of 36 vehicles that have been investigated throughout this study, surface, wake and/or underbody pressure measurements were carried out on the following eight vehicles, where an abbreviation to designate each vehicle is included in brackets:

- 2016 mid-size crossover SUV, 2.0L (CUV1);
- 2015 mid-size crossover SUV, 3.5L (CUV2);
- 2016 compact sedan, 1.8L HEV (CS1);
- 2015 compact executive sedan, 3.0L (CS2);
- 2016 mid-size luxury sedan, BEV (MS1);
- 2017 mid-size luxury sedan, 2.0L (MS2).
- 2017 minivan, 3.6L (MV1); and
- 2017 full-size pickup truck, 2.7L (PU1).

A list of the tests carried out for each vehicle, as well as the measurement techniques used for each test, can be found in Table 2 in Appendix A.

Full-scale wind tunnel testing is the ideal choice for this work as it allows production vehicles to be evaluated without the expense of numerical or physical modelling, providing repeatable test conditions and precise measurement differences from one vehicle to another. To ensure appropriate road-representative conditions, critical to the performance of aerodynamic technologies, the vehicles were tested with a moving ground belt, wheel rollers and boundary-layer suction in road-representative turbulent wind conditions.

## Test Setup and Procedures

### Wind Tunnel

**FIGURE 1:**
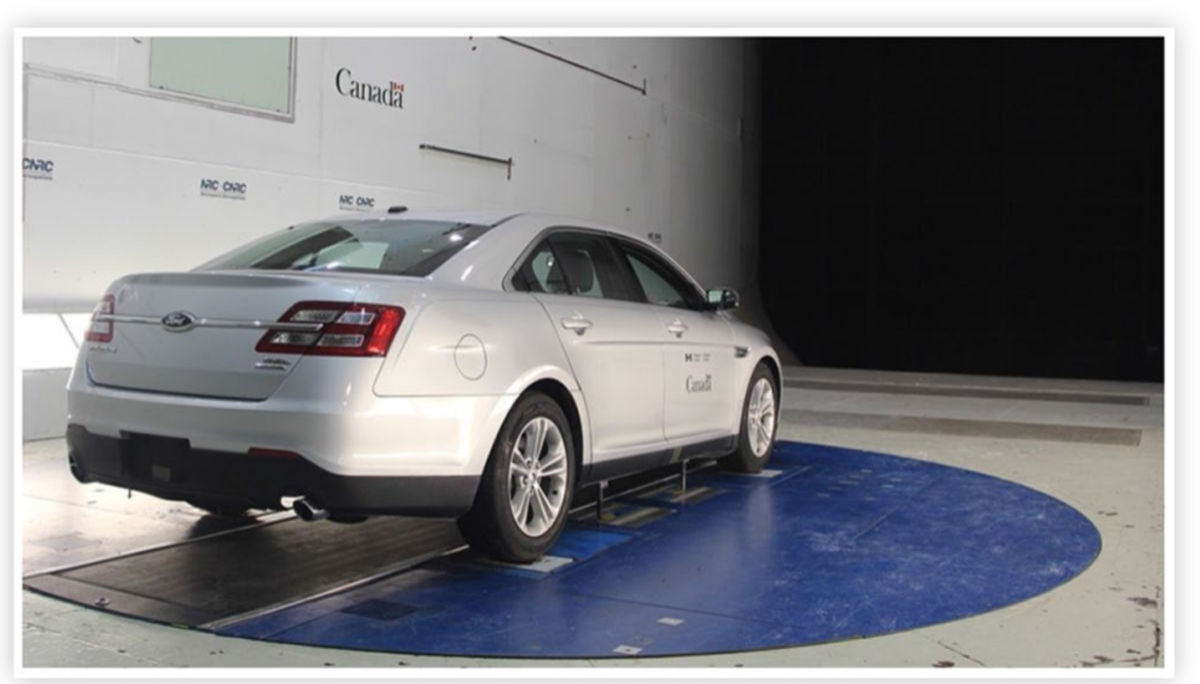
2019–01-0648 View of a full-size sedan (not tested in the current study) on the GESS in the NRC 9 m Wind Tunnel.)

The test program was undertaken in the NRC 9 m Wind Tunnel located in Ottawa, Ontario, Canada. The wind tunnel is a horizontal closed circuit atmospheric facility with a large test section (9.1 m wide × 9.1 m high × 22.9 m long) that is suitable for testing up to the largest light-duty vehicle (LDV). It is powered by an air-cooled 6.7 MW DC motor that provides a maximum wind speed of 55 m/s (200 km/h) in an empty test section. An external mechanical, pyramidal balance senses the six-components of aerodynamic forces and moments. One of the vehicles tested in an earlier phase of this study is shown in Figure 1 installed in the test section of the wind tunnel. A turntable (blue circle in Figure 1) allows rotation of the model about a vertical axis to simulate the effect of cross winds.

#### Ground Effect Simulation

The test section is equipped with a Ground Effect Simulation System (GESS) composed of a centre belt, with an exposed 1 m × 5.7 m area, four wheel rollers and four chassis struts for ride height control. Upstream of the turntable are two plenae for a distributed suction system, calibrated to reduce the displacement thickness of the floor boundary layer to 4 mm at the leading edge of the turntable, without inducing flow angularity at the model [3]. Breathers on the floor and walls at the test section exit are vented to the tunnel surroundings to equalize the otherwise negative pressure in the test section with atmospheric pressure.

#### Turbulence Simulation

A Road Turbulence System (RTS) [4], shown to be appropriate for the study of the aerodynamics of LDVs [5], was used for the experiments. In earlier phases of this study, it was concluded that flow turbulence generated by the RTS can have an impact on the performance of drag reduction technologies for LDVs [2, 1]. As such, most of the vehicles discussed in this paper were tested in road-representative turbulent flow generated by the RTS. Exceptions are one of the CUVs, which was tested in both smooth and turbulent flow, and the pickup truck, which was tested in smooth flow only.

### Vehicle Preparation and Installation

The vehicles were installed so that their front bumpers were approximately 500 mm downstream of the leading edge of the centre belt. The belt runs under the vehicle, but inboard of the wheels, as shown in Figure 1.

**FIGURE 2:**
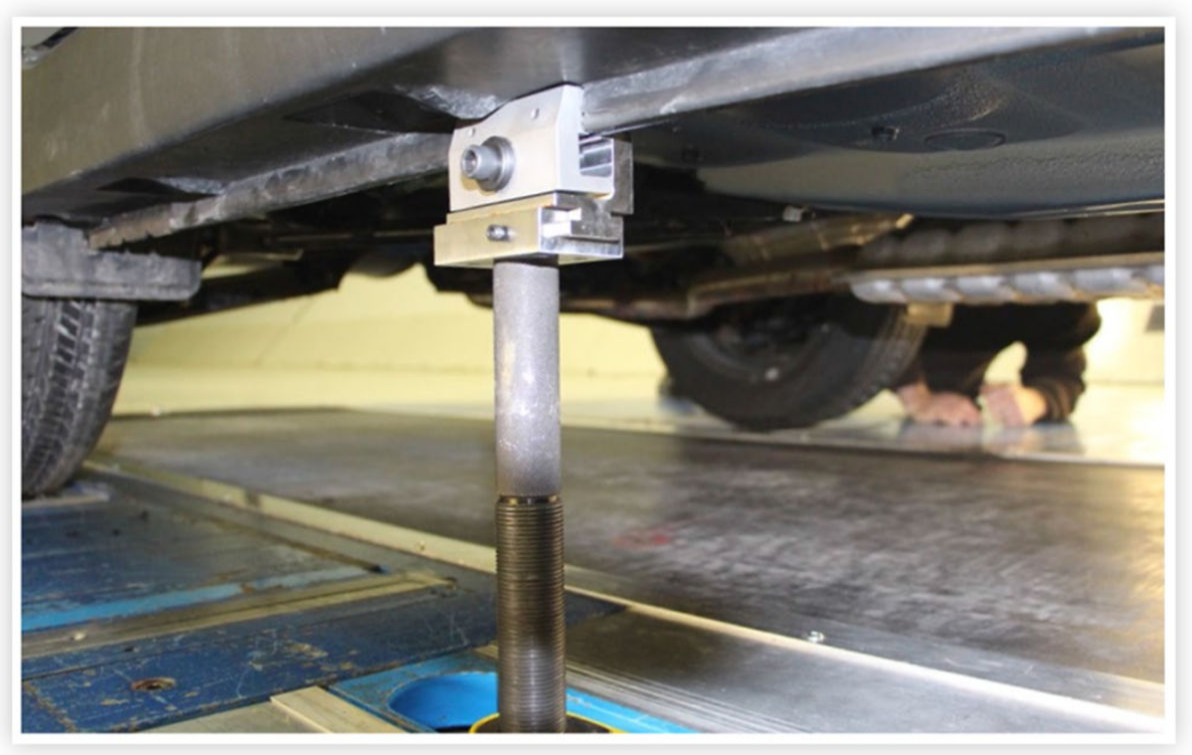
Close-up view of a rear GESS strut and rear GESS clamp attached to the rocker panel of a small SUV.

The GESS wheel rollers do not support the full weight of the vehicle, so pre-test preparations included measuring the baseline ride height and removing the suspension springs. The vehicles were mounted in the test section on four chassis struts, clamped to the rocker panels, as shown in Figure 2. The strut clamps allow for the possibility of adjusting the ride height independently between the rear and front support points.

Since the engines/motors were not operated during testing, the vehicle driveshafts were removed to prevent transmission damage from wheel rotation without oil pressure. In most cases, this was accomplished by disassembling the constant velocity (CV) joints, removing the axle, and reinstalling the CV joints to seal the differentials/transmissions and hold the wheel bearings in place.

**FIGURE 3:**
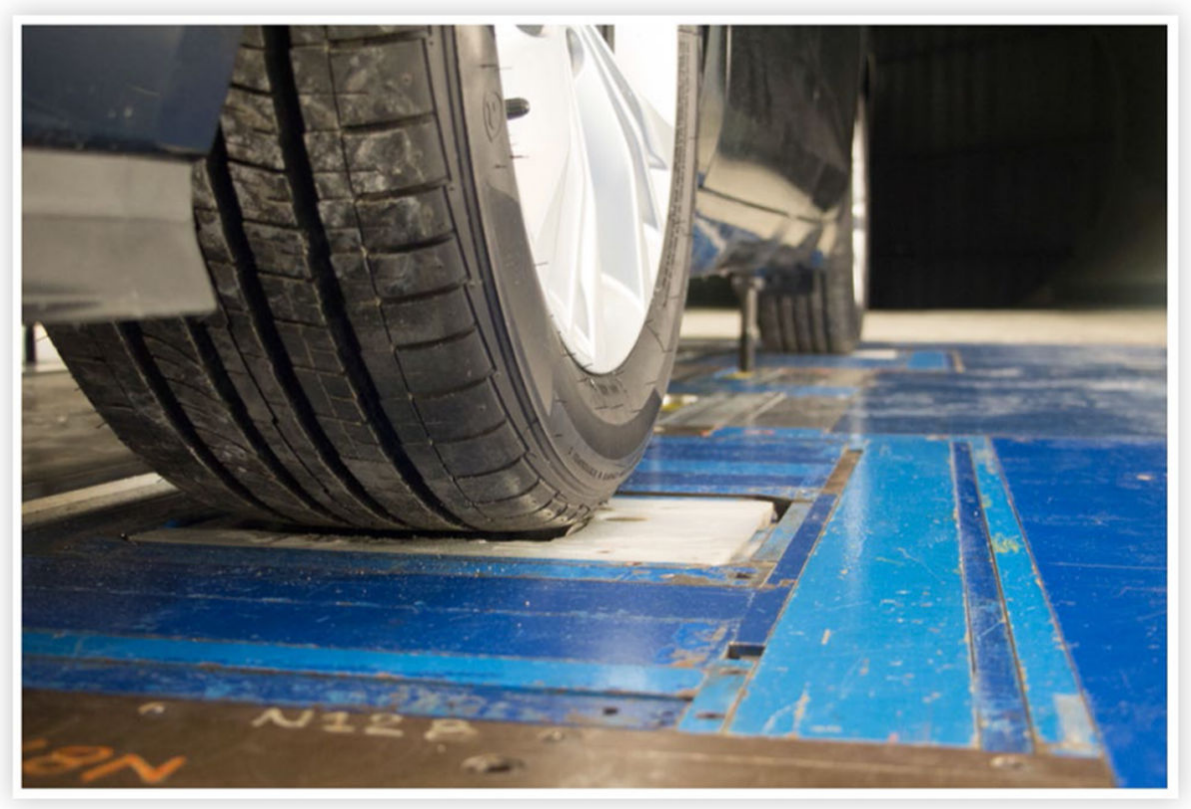
Close-up view of a LDV wheel and GESS wheel roller in the NRC 9 m Wind Tunnel.

Once the suspension springs were removed and the axles disengaged, no other modifications to the wheels and tires were necessary. The tire pressures were adjusted to the manufacturers’ specifications, and the baseline ride height was measured with 68 kg (150-lbs) of ballast on both front seats before removal of the springs. The GESS system ensured that the wheel rotation matched the wind speed. Figure 3 shows a close-up of a wheel on a roller.

### Baseline Conditions

The baseline condition describes the vehicle as received i) with all doors and windows closed and external mirrors adjusted to driving positions; ii) ride heights adjusted with the GESS struts to the baseline height as defined earlier, and iii) active grille shutters (if present) 100% closed.

### Active Grille Shutter and Cooling Drag Tests

All vehicles except the compact executive sedan (CS2) were equipped with an OEM active grille shutter (AGS) system that could be 100% closed and 100% opened. An electronic system was devised so that the AGS actuator could be controlled remotely.

In order to estimate the maximum potential benefit of AGS systems, measurements were also made with the external grille of each vehicle completely sealed with tape.

### Ride Height Tests

Because the vehicles in this study were supported by chassis struts and the suspension springs were removed, a ride height matrix could be run without further preparation. Reductions from 20 mm to up to 40 mm were tested, depending on the lowest possible configuration for each vehicle. To determine the effect of having ride height control on one axle only, thus saving cost and weight, the test matrix included some pitch-change configurations.

### Wheel Dam Tests

Surface pressure results were obtained for a mid-size sedan with and without its front wheel dams to correlate with the drag reduction performance of this technology. The wheel dams of this vehicle were contoured and likely designed to reduce wheel drag, which contributes significantly to overall drag.

### Underbody Treatments (OEM and Idealized Prototypes)

Underbody panels shield bluff underbody components from high-energy air. Their use on vehicles has been increasing over the last few model cycles as fuel consumption targets become stricter. OEM underbody panels were removed so that their effect on drag, surface, wake and underbody pressures could be evaluated for four vehicles of this study: a crossover SUV, two compact sedans and a mid-size sedan.

**FIGURE 4:**
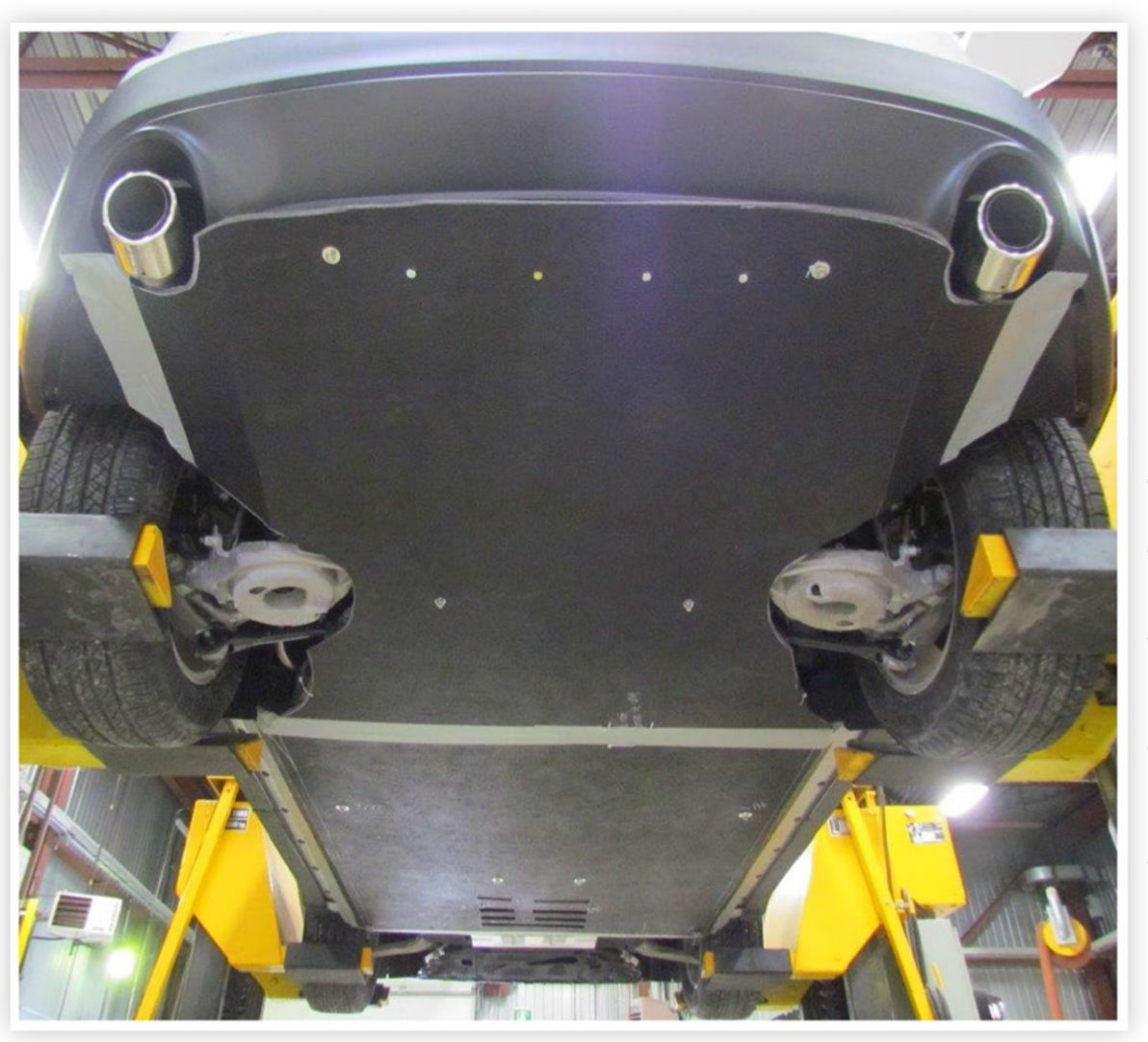
Rear view of prototype, smooth underbody panels on a vehicle.

To quantify the maximum potential benefits of a fully smooth underbody panel package without considering weight or cooling trade-offs, the crossover SUV and one of thecompact sedans tested above were equipped with custom panels to smooth the underbody surfaces not covered by the OEM panels. An example is shown in Figure 4. As the front and outer-middle OEM underbodies of these two vehicles were already smooth, the custom panels were used to smooth the center-middle and rear underbody. The prototypes allowed for full suspension motion but partially covered the exhaust system, so are not practical designs and would need to be optimized for on-road use.

### Wheel Curtains

Three vehicles of this study were equipped with wheel curtains, a device designed to guide the flow of air around the front wheels in order to reduce wheel drag. Oncoming air is channelled into ducts located below the headlamps, near the outer edges of the front bumper, through to openings in the front wheel wells, where it is directed across and parallel to the outer surface of the wheel and tire. The aim is thus to suppress the wake generated by the wheels. The effect of this device was assessed by sealing the air curtain ducts with tape. The tests were done at different ride heights, since this parameter has a considerable effect on the wheel drag.

### Side Mirrors

Although not yet permitted by regulations, side mirrors can be replaced by less aerodynamically-intrusive cameras to reduce drag. To investigate the maximum potential drag benefit, and the corresponding effect on surface and wake pressures, six vehicles were tested with and without their side mirrors installed: one crossover SUV, all four sedans and the pickup truck. After removing the side mirrors, any resulting cavity was smoothed with tape.

### Data Acquisition and Test Procedures

#### Wind Speed and Aerodynamic Force Measurements

The data acquisition system was configured for a sampling time of 40 seconds per test condition at a sampling rate of 20 Hz. Data sampling was initiated when the flow conditions were stabilized. The recorded time series data were averaged over this sampling period. Based on an analysis of random and bias measurement uncertainties, all variations of drag of 0.5% or more are considered as statistically significant in this study.

Wind-tunnel testing occurs in a current of air with finite boundaries (walls and ceiling) that influence the flow around the model as it blocks part of this current. This contrasts real driving conditions where the air is free to expand around the model without interference from nearby walls or ceiling. Though the vehicles in the current investigation were relatively small compared to the test section area, corrections using the Maskell III [6, 7] blockage-correction technique were carried out for all test runs. For vehicles at zero yaw, the maximum adjustment to velocity and drag area was 4% and 8%, respectively, and corresponded to the pickup truck, which was the largest test vehicle.

Since precise frontal area data were not available, absolute drag coefficients as typically reported in automotive publications are not presented in this paper. Instead, the results are presented in terms of *C_D_A* (drag area in m^2^) or percentage changes of drag area with respect to the *C_D_A* of the baseline configuration. *C_D_A* offers a more complete understanding of aerodynamic resistance as it captures the effect of frontal area. Frontal area is equally as important as *C_D_*, which can be thought of as a description of the efficiency of the vehicle shape. While manufacturers have generally been lowering *C_D_* as a model evolves, they have often simultaneously increased vehicle size, offsetting the efficiency gains to some degree. Any discussion of vehicle design, with the aim of reducing aerodynamic drag, must consider frontal area as it directly impacts and is the most easily changed parameter of drag force.

#### Surface Pressure Measurements

Surface pressures were measured by installing up to 40 surface pressure disks at strategic locations on each of the four sedans and two crossover SUVs. The aluminum disks, 25 mm in diameter, had a static pressure tap drilled into the center and a small tubulation on the side that was connected via flexible tubing to a compact electronic pressure scanning module located inside the vehicle. Surface pressures were sampled at 120 Hz for 40 seconds at each test condition. Measurements acquired with and without the surface pressure disks and tubing installed showed that this equipment had a negligible effect on the drag measurements.

#### Wake and Underbody Pressure Measurements

Using a 2 m-high rake of 41 equally-spaced total pressure probes, vehicle wake pressures were mapped in two cross-flow planes located from 0.1 to 0.4 vehicle lengths, *L*, downstream of the rear bumper. Wake pressures were measured for all test vehicles at baseline conditions. A smaller rake of 4 to 6 total pressure probes was used to map underbody pressures in a cross-flow plane located between the rear wheel and rear bumper. The underbody pressures were measured at baseline conditions for a crossover SUV, a compact sedan and a mid-size luxury sedan. The wake and/or underbody pressure surveys were also performed for some non-baseline cooling, underbody, ride height, wheel curtain and side mirror configurations. The rakes were traversed laterally along 3-m wide tracks in steps of 100 mm. When both rakes were used, the rakes were offset by 800 mm in the spanwise direction in order to minimize the effects of flow interference on the wake rake measurements due to the presence of the underbody rake. The wake and underbody rake pressures were sampled at 120 Hz for 15 to 30 seconds per spanwise position using compact electronic pressure scanning modules.

For all aerodynamic load measurements, both rakes were stationary and offset by 1.5 m from the vehicle centerline. In order to assess any possible effects of the rakes and tracks on aerodynamic drag measurements, baseline measurements were obtained with and without the underbody rake and track installed, with the wake rake at different streamwise locations and with the wake rake and track completely removed. The corresponding variation in drag measurements was 0.4% or less, which is within the uncertainty of the measurements. The baseline drag measurements reported in this study were acquired without the underbody rake installed and with the wake rake in its most downstream position. Delta drag area measurements for the various aerodynamic treatments were calculated based on a reference condition with the same wake and underbody rake configuration.

## Results and Discussion

### Baseline Surface Pressures

The surface pressure results are presented in the form of the surface pressure coefficient *C_PS_*, defined as:

(1) $$C_{PS}=\frac{\left(P_{S,\;surface\;}-P_{S,\;freectraan\;}\right)}{Q_{fressiream\;}}$$

where *P*_*S*, *surface*_ is the static pressure on the vehicle surface, and *PS*, *freestream* and *Qfreestream* are the tunnel freestream static and dynamic pressures, respectively.

Surface pressure taps were distributed at various locations on the test vehicles:

- centerline of front bumper/grille surfaces (two sedans also had taps near the headlights);
- centerline of hood (only on two sedans);
- roof aligned with A-pillar (on only 2 sedans), B-pillar and C-pillar;
- trailing edge of roof/upper trunk surface of CUVs/ sedans;
- two or three spanwise rows on rear window;
- two or three spanwise rows on rear trunk and bumper;
- side surface of rear taillights (some vehicles had additional side surface taps); and
- underbody and/or rear surface of rear diffuser (only on some vehicles).

**FIGURE 5:**
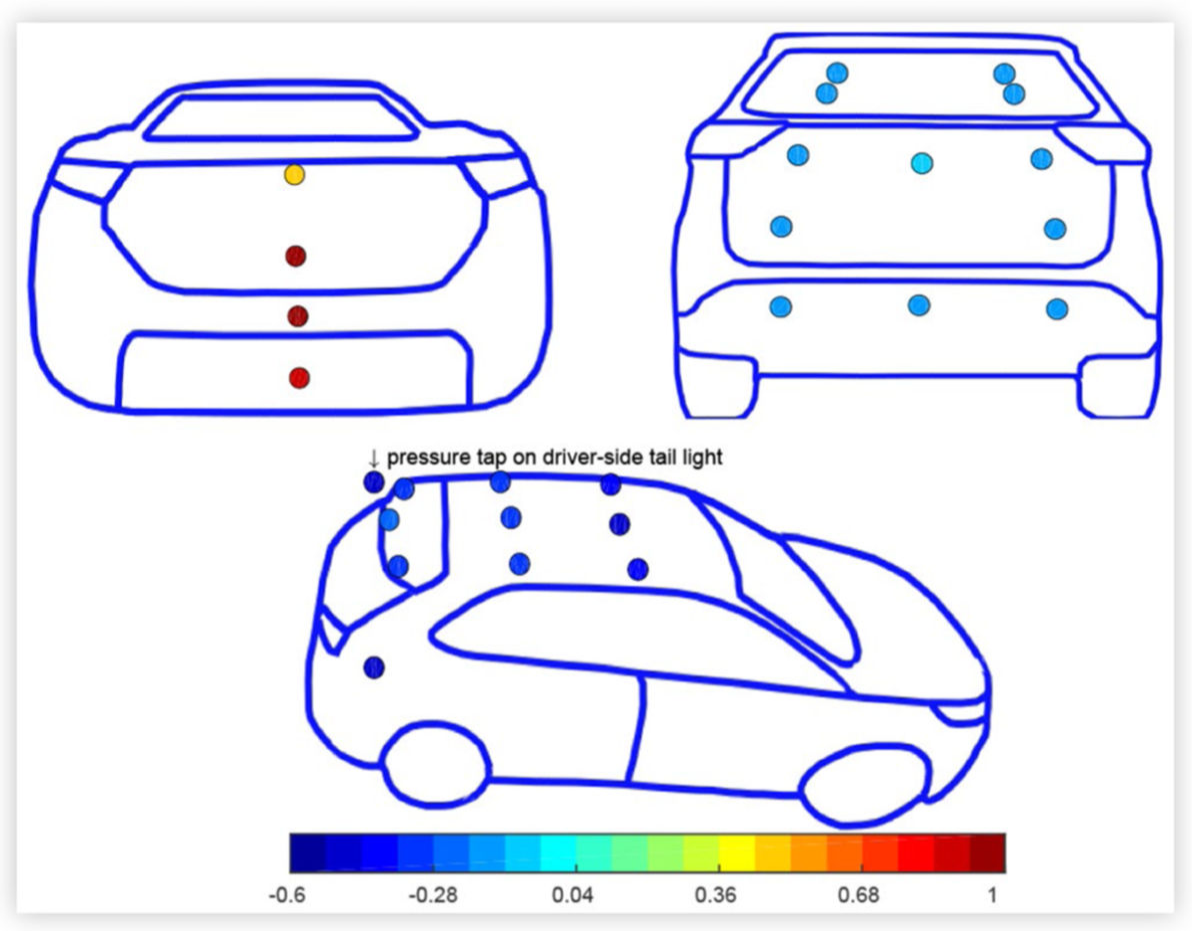
Surface pressure coefficients *C*_*PS*_ on a CUV in the baseline configuration at ° yaw in turbulent flow at 110 km/h.

**FIGURE 6:**
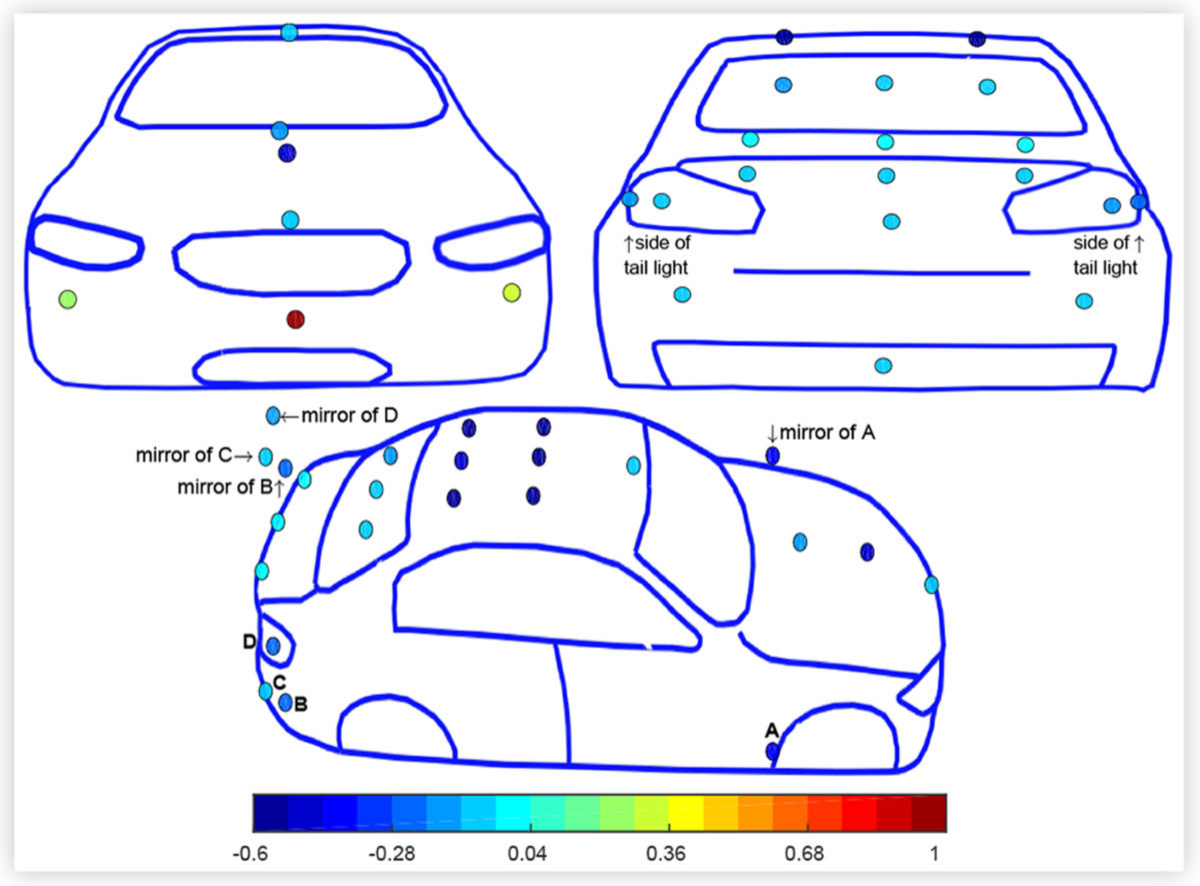
Surface pressure coefficients *C*_*PS*_ on a sedan in the baseline configuration at 0° yaw in turbulent flow at 110 km/h.

Typical results are shown for a CUV and a sedan in the baseline configuration at 0° yaw in turbulent flow in Figures 5 and 6. *C_PS_* values are plotted as colormap values at the pressure tap locations. From a value of 1 near the stagnation point at the front of the vehicles, *C_PS_* typically decreased to negative values as the flow accelerated over the front of the hood before increasing towards the windshield. The surface pressure reached a minimum near the highest point on the roof and increased as the flow expanded towards the rear (base) of the vehicle. The surface pressure distributions were generally symmetric about the centreline of the vehicles.

**FIGURE 7:**
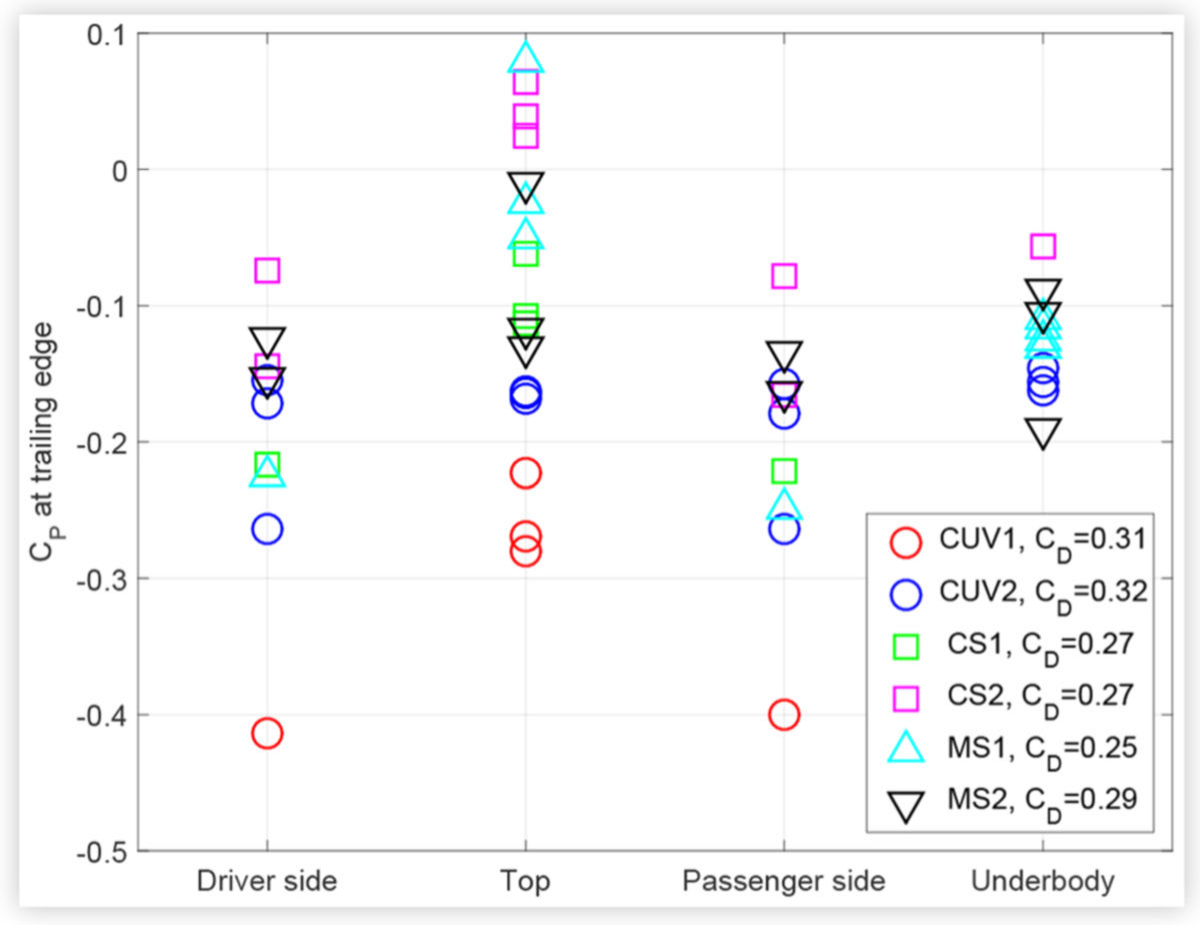
Trailing edge surface pressure coefficients *C*_*P*_ for the test vehicles.

Designing a vehicle so that the trailing edge surface pressures around the periphery of the vehicle are relatively uniform has been thought to reduce drag by equalizing as much as possible the velocity of the airflows that meet at the back end of the vehicle [8]. This in turn would minimize the strength of the shear layer and resulting vortices that induce drag. Figure 7 shows the surface pressures near the trailing edge of the test vehicles on the side, upper and lower surfaces of the vehicles. Not all vehicles had underbody trailing edge taps. Taps were distributed fairly evenly across the trailing edge surfaces on which more than one tap was installed. The drag coefficient *C_D_* measured for each vehicle is indicated in the legend for comparison, where the manufacturer-specified width times the height was used as the vehicle frontal area in the calculation of *C_D_*. CUV2 had the most uniform trailing edge pressures. However, for each class of vehicle, there does not seem to be an obvious correlation between uniformity of trailing edge pressures and drag coefficient.

## Effect of Drag Reduction Technologies on Surface Pressures

### AGS and Cooling Treatments

**FIGURE 8:**
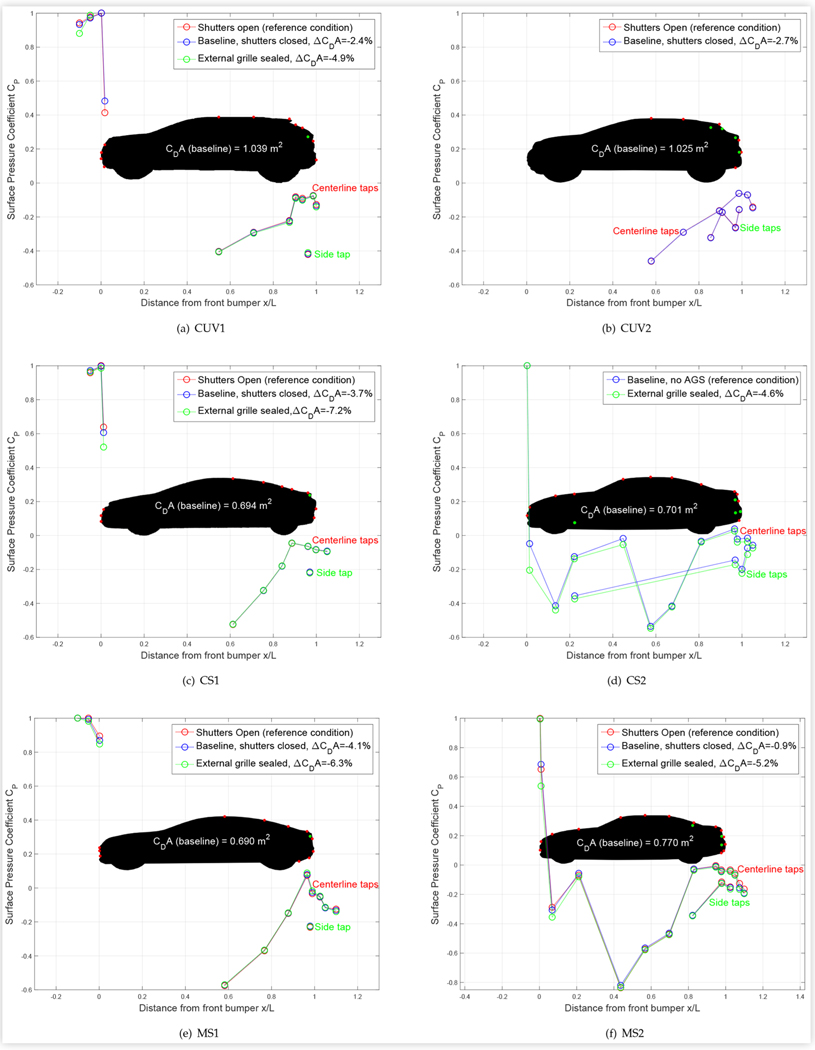
Centerline and driver-side surface pressure coefficients *C*_*P*_ for the test vehicles at 0° yaw in different cooling configurations. Results from lowest taps are sometimes plotted offset to the left or right on the *x*/*L* axis for clarity.

Centerline surface pressure distributions provide a quantitative representation of results that highlights differences due to the technologies examined. Figure 8 shows the surface pressure coefficients along the centerline of the test vehicles in various cooling configurations: baseline, shutters open (where equipped) and with the external grille sealed. The locations of the centerline taps are indicated by the red dots on the schematic side profile of the vehicle. Results from the driver side surface taps were also included. Side tap locations are indicated by the green dots on the schematic. Results from the lowest front and rear centerline taps and side taps are sometimes plotted offset to the left or right on the *x*/*L* axis, rather than at their true position, for clarity. The percentage change in drag area *C_D_A* (where *A* is the vehicle frontal area) with respect to the indicated reference condition is shown in the legend of the figure. Changes in surface pressure coefficients beyond ±1% of the freestream dynamic pressure, *Q_freestream_*, were considered to be significant and well above the normal run to run variation.

For most vehicles, changing the cooling configuration affected the surface pressures measured on the front bumper and grille surfaces. The biggest changes were at the highest tap on the front surface. For all the sedans, sealing the external grille significantly lowered the surface pressure, which corresponds to an increase in flow speed, at this location. A similar result was shown by Hupertz et al. [9] for the Open Cooling DrivAer (OCDA) model with the upper and lower grille openings blanked. For 2 of the 3 sedans equipped with AGS, closing the shutters also lowered the the surface pressure at the uppermost front tap, although to a lesser extent than sealing the grille. This is to be expected, since sealing the grille approximates an ideal closed shutter.

When comparing the sealed grille and closed shutter results, the relatively small changes in front surface pressure and drag area reduction for MS1 can be explained by the fact that the AGS of MS1 are located very close to the front grille. This typically increases the effectiveness of the AGS by providing a better approximation to an external seal.

For MS2, closing the shutters increased the pressure at the uppermost front tap, but caused a small decrease in pressure at the tap at the front of the hood. Sealing the grille significantly decreased the pressure at both of these locations on MS2 as well as on CS2. Restricting the cooling flow thus increases the air speed over the hood, as the incoming flow is redirected towards the upper surface rather than entering the engine bay.

The surface pressure changes at the front of CUV1 due to blocking the cooling flow were different than for the sedans. Closing the shutters increased the pressure at the highest front tap (which was not accessible with the grille sealed), and sealing the grille decreased the pressure at the lowest front tap. CUV2 was not equipped with front surface taps.

Closing/sealing the grille openings caused small to negligible changes in the rear centerline and side surface pressures of the two CUVs, CS1 and MS1. It caused more consistent reductions at most centerline and side tap locations on CS2, as well as on the hood, front of the roof and lower rear and side surfaces of MS2. Although not shown in the figures, pressures measured near the headlights of CS2 and MS2 were also significantly lower due to restricting the cooling flow, by up to 5% of the freestream dynamic pressure due to closing the AGS of MS2, and by more than double this amount due to sealing the grille. The changes are consistent with the cooling flow being redirected above as well as around the sides of these vehicles. Sealing the external grille redirects the flow more efficiently than closing the AGS, as it reduces momentum losses due to flow entering the cavity between the grille and closed shutter surfaces, and results in a smaller area of high pressure on the front face of the vehicle.

Figure 8 shows that closing and/or sealing the cooling systems significantly lowered the measured centerline base pressures of CS2 and MS2, as observed by Hupertz et al. [9] for the OCDA model as well as by Pitman and Gaylard for an SUV [10], but had less to no effect on those of the CUVs, CS1 and MS1. This could explain the smaller amount of drag reduction measured for CS2 and MS2 compared to the other vehicles, as the reduction in cooling drag was somewhat countered by an increase in base drag for CS2 and MS2, whereas the cooling drag reduction technologies of some of the other vehicles may have been more effectively optimized. In fact, closing and sealing the cooling system of MS1 slightly raised the upper base pressure, enhancing the drag reduction.

### Ride Height Control

**FIGURE 9:**
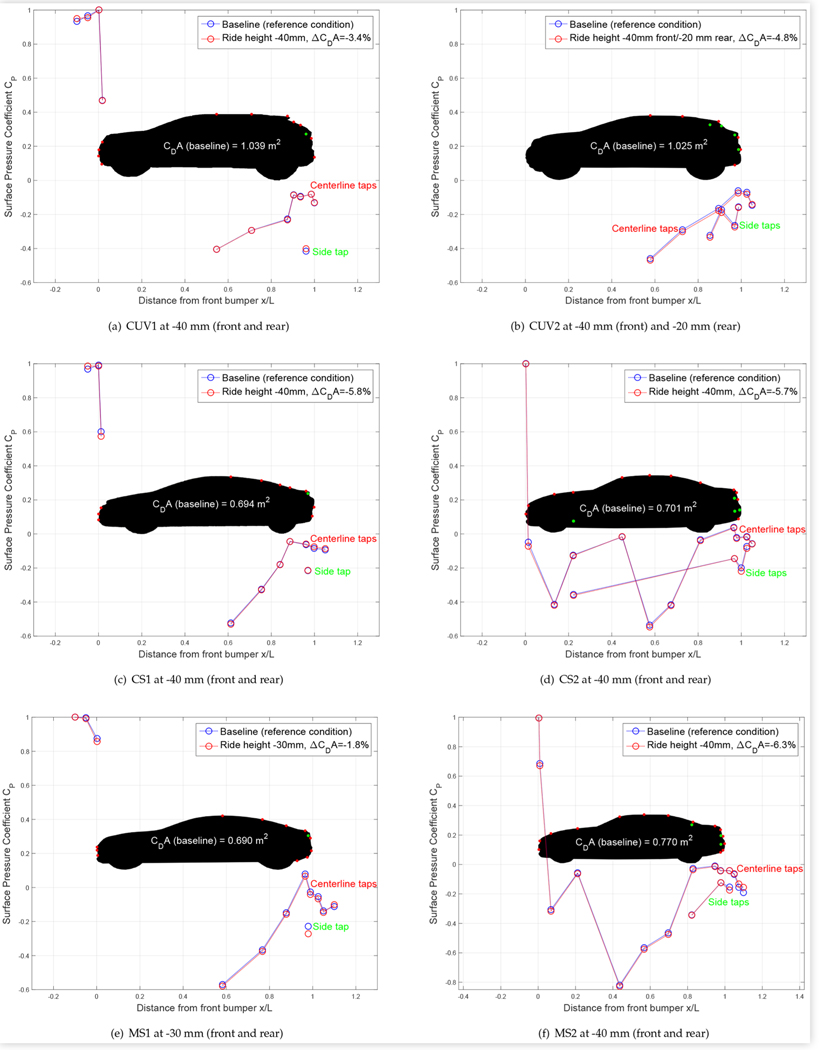
Centerline and driver-side surface pressure coefficients *C*_*P*_ for the test vehicles at 0° yaw at baseline and reduced ride height. Results from lowest taps are sometimes plotted offset to the left or right on the *x*/*L* axis for clarity.

Figure 9 shows the surface pressure coefficients at the centerline and driver side taps of the test vehicles in the baseline configuration and at the lowest drag ride height configuration that was tested. For all vehicles except CUV2 and MS1, this was a reduction of 40 mm at the front and rear of the vehicle. For CUV2, it was a front and rear ride height reduction of 40 mm and 20 mm, respectively. For MS1 it was a front and rear ride height reduction of 30 mm as it was not possible to lower the vehicle any further. The location of the pressure taps and the resulting reduction in *C_D_A* is indicated in the same way as in Figure 8.

Lowering the ride height reduces drag area by reducing the exposed frontal area of the wheels. However, it can also redistribute the flow around the vehicle, causing more air to flow above and less air to flow below the vehicle, where it can impinge on rough underbody surfaces and the wheels to increase drag. Changing the ride height can allow the flow to approach the body and wheels of the vehicle from a more favorable angle, reducing losses. The surface pressure results are generally consistent with this, with lower pressures on the upper and side surfaces of the test vehicles, indicating higher velocities, and slightly higher pressures, where measured, on underbody surfaces.

As opposed to changing the cooling configuration, lowering the ride height had less of an effect on the measured surface pressures of CUV1 compared to CUV2. The only significant changes on CUV1 were slight increases at the lowest front tap and at the side tap, whereas there were measurable decreases at most upper and rear-facing centerline taps and at the three highest side taps of CUV2. Corresponding drag reduction was also more significant for CUV2 than for CUV1, despite the reduction in base pressure on the former.

For the compact sedans, lowering the ride height reduced the surface pressure at the uppermost front tap by a comparable amount. The only other significant changes were an increase in pressure at the lowest front tap of CS1 and decreases in pressure at the highest roof tap and the lowest side taps of CS2. The level of drag reduction was similar for both CUVs.

The surface pressure changes at the upper front taps of the mid-size sedans were similar but slightly less pronounced than those on the compact sedans. However, there were significant decreases in pressure at the rear/lowest side taps of both vehicles, particularly for MS1, suggesting that more air flowed around the sides of these lowered vehicles. Pressures on the rear diffuser of both mid-size sedans were generally higher, consistent with a decrease in flow under the vehicles due to lowering the ride height. Lowering the ride height decreased the centerline base pressures of MS1 but had no significant effect on those of MS2, which is perhaps related to the higher drag reduction observed for MS2.

### Production and Custom Underbody Panels

**FIGURE 10:**
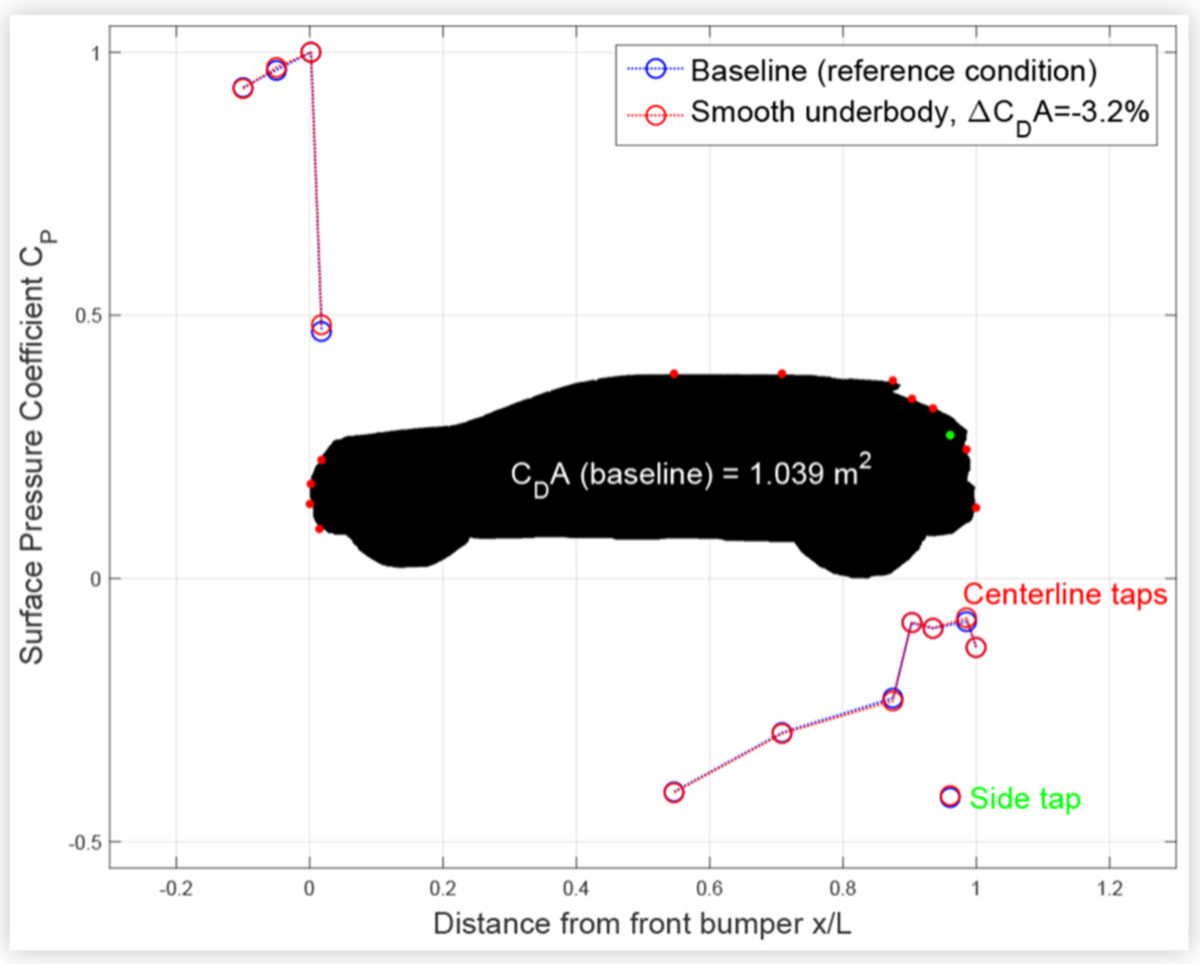
Centerline and driver-side surface pressure coefficients *C*_*P*_ for CUV1 at baseline and with a custom smooth underbody installed. Results from the two lowest front taps are plotted offset to the left on the *x*/*L* axis for clarity.

Smoothing the underbody with custom panels caused a small but measurable increase in pressure at the highest front tap of CUV1, as shown in Figure 10, but no significant changes in the measured surface pressures on CS1. The corresponding 1.6% reduction in drag area for CS1 was half as much as for CUV1. This is likely because the underbody of the compact sedan was already fairly smooth, whereas there was more room for improvement on CUV1.

**FIGURE 11:**
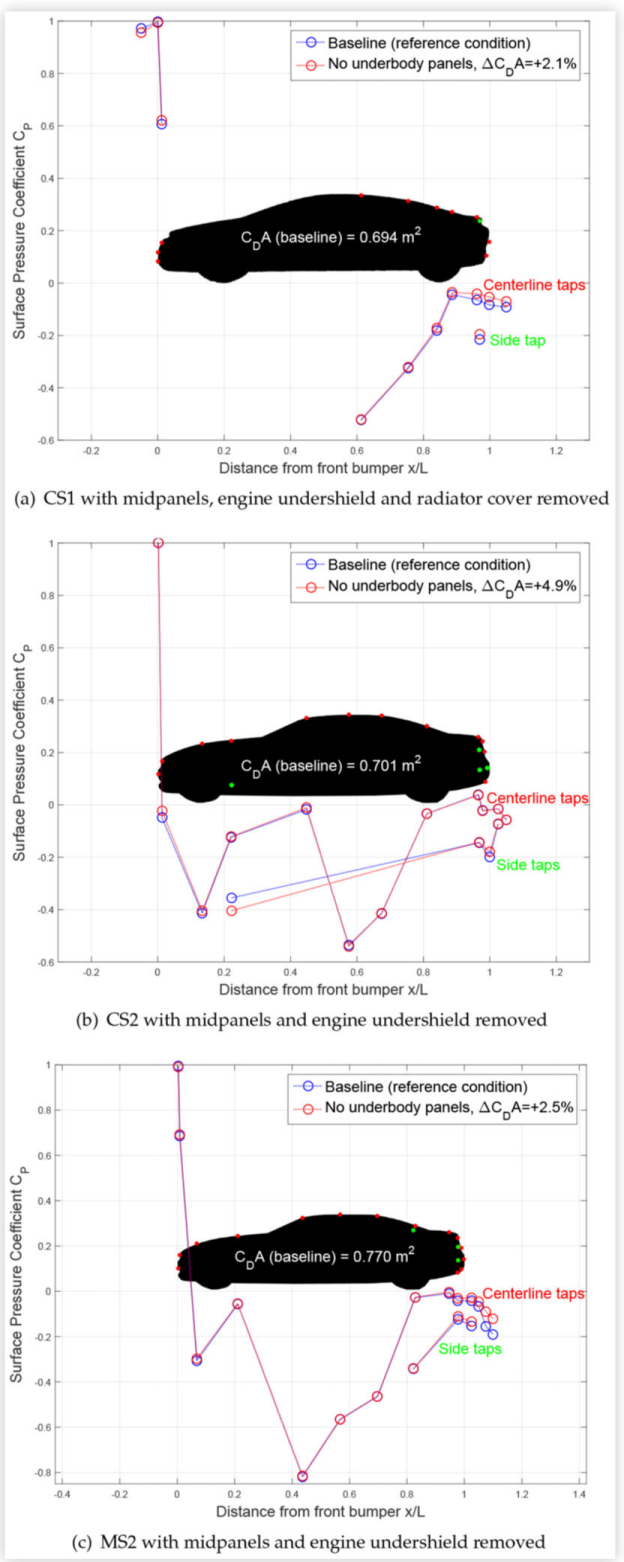
Centerline and driver-side surface pressure coefficients *C*_*P*_ for three sedans at baseline and with OEM underbody panels removed. Results from lowest taps are sometimes plotted offset to the left or right on the *x*/*L* axis for clarity.

Removing the OEM underbody panels of CUV1 caused negligible changes in the measured surface pressures. The corresponding 2.5% increase in drag area was thus likely due to local drag on rough underbody components. The largest surface pressure changes in the underbody panel study were measured for the three sedans with their OEM underbody panels removed, as shown in Figure 11. The OEM midpanels and engine undershield was removed from all vehicles. CS1 was equipped with an underbody radiator cover, which was also removed.

The lower pressure at the lowest front tap of CS1 along with the higher pressure at the highest front tap of both compact sedans suggest that more air flowed under the vehicle with the OEM panels removed, increasing underbody drag. The higher centerline upper and side surface pressures at the rear of CS1 suggest that less air flowed around and above the vehicle due to the flow being redirected below. It appears, therefore, that the underbody panels of CS1 decrease underbody drag but cause changes that increase base drag, which may explain the relatively small 2.1% increase in overall drag due to removing them.

For the CS2, significant surface pressure changes at the taps downstream of the wheels, particularly the front wheels, indicate that removing the underbody panels modified the wheel aerodynamics such that the relatively large 4.9% increase in total drag may be related to increased wheel drag.

Finally, the increased surface pressures at the rear centerline and side taps of MS2 suggests a change in the flow of air into the base region due to removing the underbody panels. These changes led to a 2.5% increase in overall drag.

All of these results reflect the fact that surface pressure taps must be strategically placed to capture changes in surface pressure, as were the taps on the front fenders of CS2 and on the rear underbody diffuser of MS2.

### Wheel Curtains and Wheel Dams

Of the vehicles equipped with wheel curtains, only those of the compact sedan reduced drag significantly. The effect of the wheel curtains were evaluated by sealing their inlet and outlet with tape at baseline and reduced ride height. With the ride height reduced by 40 mm, blocking the wheel curtains of the compact sedan increased the drag area by 2.1%, but the only significant change in surface pressure was an increase equivalent to 1.7% of the freestream dynamic pressure on the front fender just downstream of the wheel. This is consistent with a stronger front wheel wake due to blocking the wheel curtains. Blocking the wheel curtains at baseline ride height increased the drag area by roughly half of the amount measured at reduced ride height, and caused measurable surface pressure changes only at centerline taps at the leading edge of the hood and at the highest point on the roof, where the pressure was reduced by amounts equivalent to 1.5% and 1.1%, respectively, of the freestream dynamic pressure. This is consistent with the flow being redirected from the wheel curtain inlets near the headlights to the upper surface of the vehicle.

Removing the contoured front wheel dams of the mid-size sedan caused measurable increases in surface pressure at centerline taps on the upper part of the front grille and near the leading edge of the hood that were equivalent to 1.4% and 1.6%, respectively, of the freestream dynamic pressure. There were larger pressure increases, of up to 3% of the freestream dynamic pressure, at taps located near the front headlights. Changes at all other taps on the vehicle were within the uncertainty of the measurements. The results suggest that removing the wheel dams, by allowing more air to flow around the wheels, caused less air to flow above and around the sedan. This caused a 4.5% increase in overall drag, which was likely due to increased wheel and underbody drag.

### Side Mirror Removal

Seven vehicles were tested with their side mirrors removed over the course of the wind tunnel study, five of which were equipped with pressure taps. Although reductions in zero-yaw drag area of 2.4% to 3.9% were measured for all vehicles, the pressure taps were not always optimally placed to detect changes in surface pressures. Significant changes in pressure were detected only on the side surface of the rear tail lights of CUV1, CS1 and MS1 and on the uppermost front tap as well as the most upstream hood tap of MS2. There were no significant changes in any of the measured surface pressures on CS2. Table 1 lists the changes in measured surface pressures along with the corresponding changes in drag area due to removing the side mirrors. It can be seen that the magnitude of the measured changes in pressure were likely related to tap placement rather than to the amount of drag reduction.

### Relating Base Pressure and Drag Measurements

**FIGURE 12:**
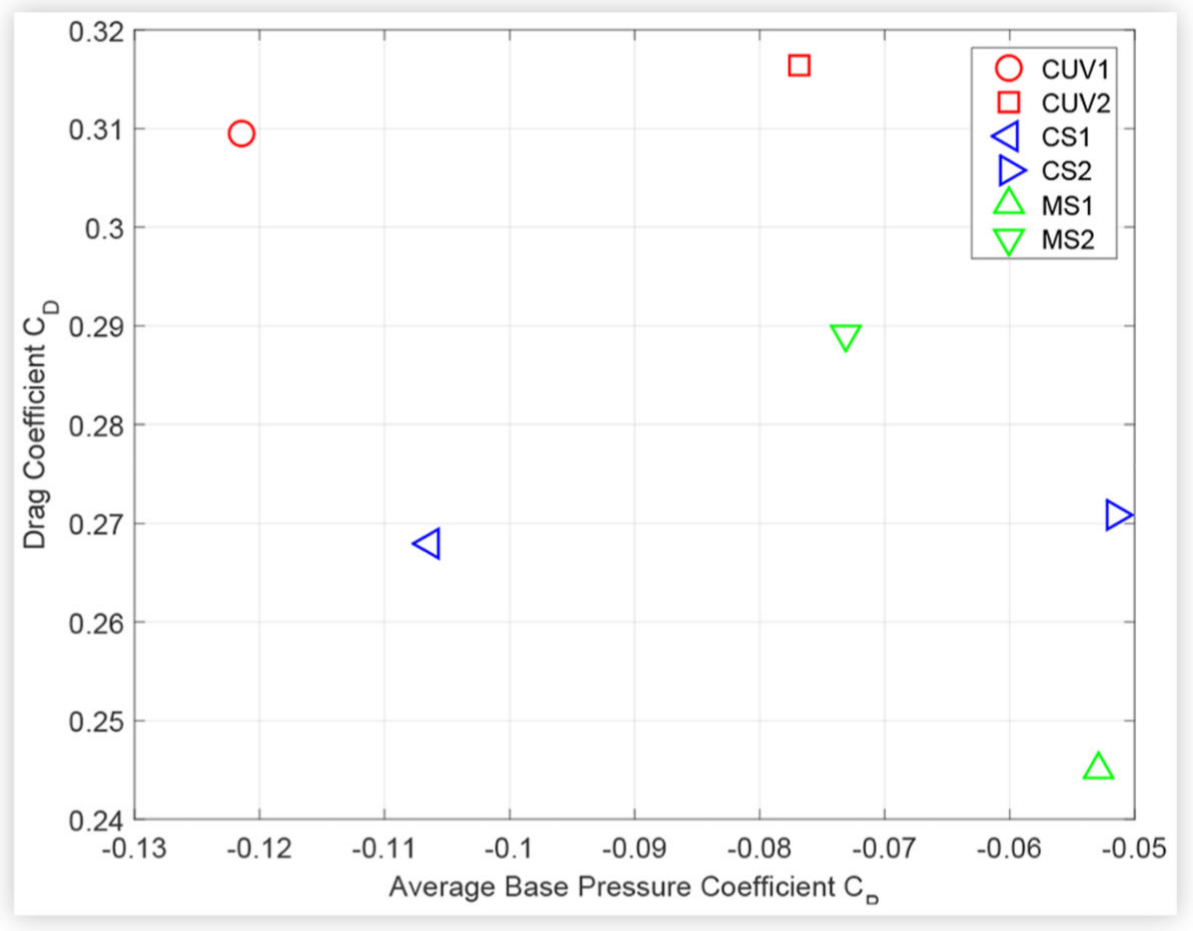
Drag coefficient *C*_*D*_ versus average base pressure coefficient *C*_*P*_ for all test vehicles at baseline conditions.

A method was devised to examine the relationship between changes in surface pressure and drag due to different technologies. The pressure on the base of a vehicle accounts for a major proportion of the overall drag. This proportion can exceed 30% for a sedan [11] and was roughly approximated to 40% for the CUV shown in Figure 5 by averaging the measurements from the rear-facing pressure taps and assuming that this average pressure acts on an area equivalent to the frontal area. Consequently, the average base pressure was considered to be a simple and useful metric to compare with drag measurements. The average base pressure was estimated as the mean of all pressures measured on the rear-facing trunk and bumper surfaces, excluding the rear window. Measurements from at least 8 taps were averaged for all vehicles except CUV2 and MS1, for which 4 and 5 taps were used, respectively. Figure 12 shows the average base pressure versus the drag coefficient measured for all test vehicles at baseline conditions. The frontal area used to calculate the drag coefficient was taken as the width (not including side mirrors) multiplied by the height of the vehicle.

**FIGURE 13:**
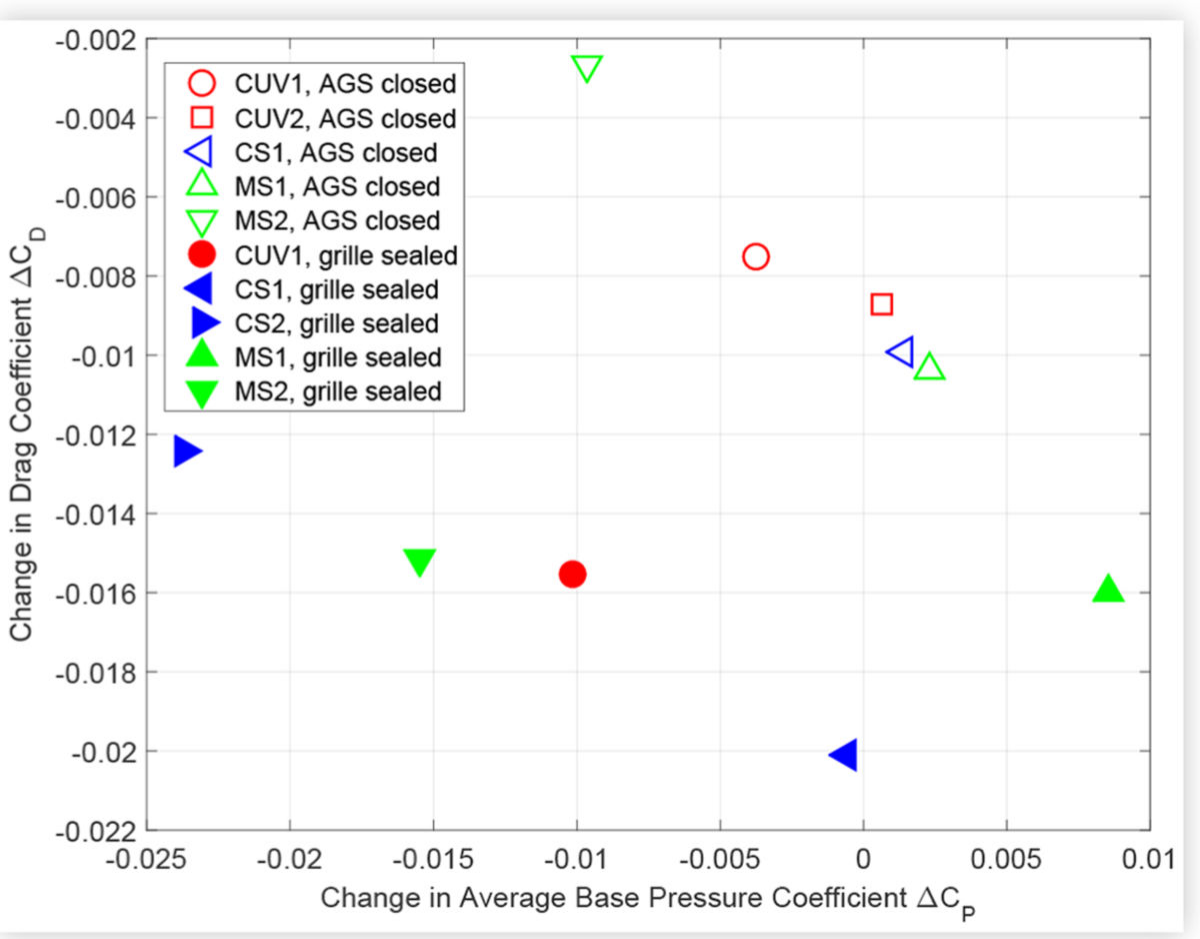
Change in drag coefficient *C*_*D*_ versus change in average base pressure coefficient *C*_*P*_ due to closing the AGS (open symbols) and sealing the external grille (closed symbols) of the test vehicles compared to open cooling conditions.

There is no obvious trend between the absolute values of *C_D_* and average base pressure plotted in Figure 12. However, Figure 13 shows a clear inverse relationship between the changes in average base pressure and drag coefficients due to closing the AGS, and a similar trend due to sealing the grille, with respect to the open cooling configuration. A comparable trend relating larger negative changes in base pressure with lower cooling drag has been reported in the literature [10], but only for different configurations of the same vehicle.

The data for closing the AGS and sealing the grille fall on two distinct lines in Figure 13 except for the point corresponding to sealing the grille of MS1, which falls on the same line as the AGS data. This anomaly could be due to the small number of taps used to calculate the average base pressure of MS1. However, Figure 13 shows that sealing the grille shifted the data for the internal combustion engine (ICE) vehicles down, towards higher drag reduction, and to the left, towards more negative base pressure, whereas the data point for the battery electric vehicle (BEV), MS1, was shifted down and to the right. The data point for the hybrid electric vehicle (HEV), CS1, fell between these two cases, shifting down but less to the left than the ICE vehicles. Thus the different cooling requirements of the HEV and BEV, which have smaller grilles than the ICE vehicles, may have had an influence on the observed trends. The efficient design of the BEV AGS, confirmed by the smaller additional drag reduction due to sealing the grille, suggests that the increase in base pressure due to blocking the cooling flow may have been specifically designed.

**FIGURE 14:**
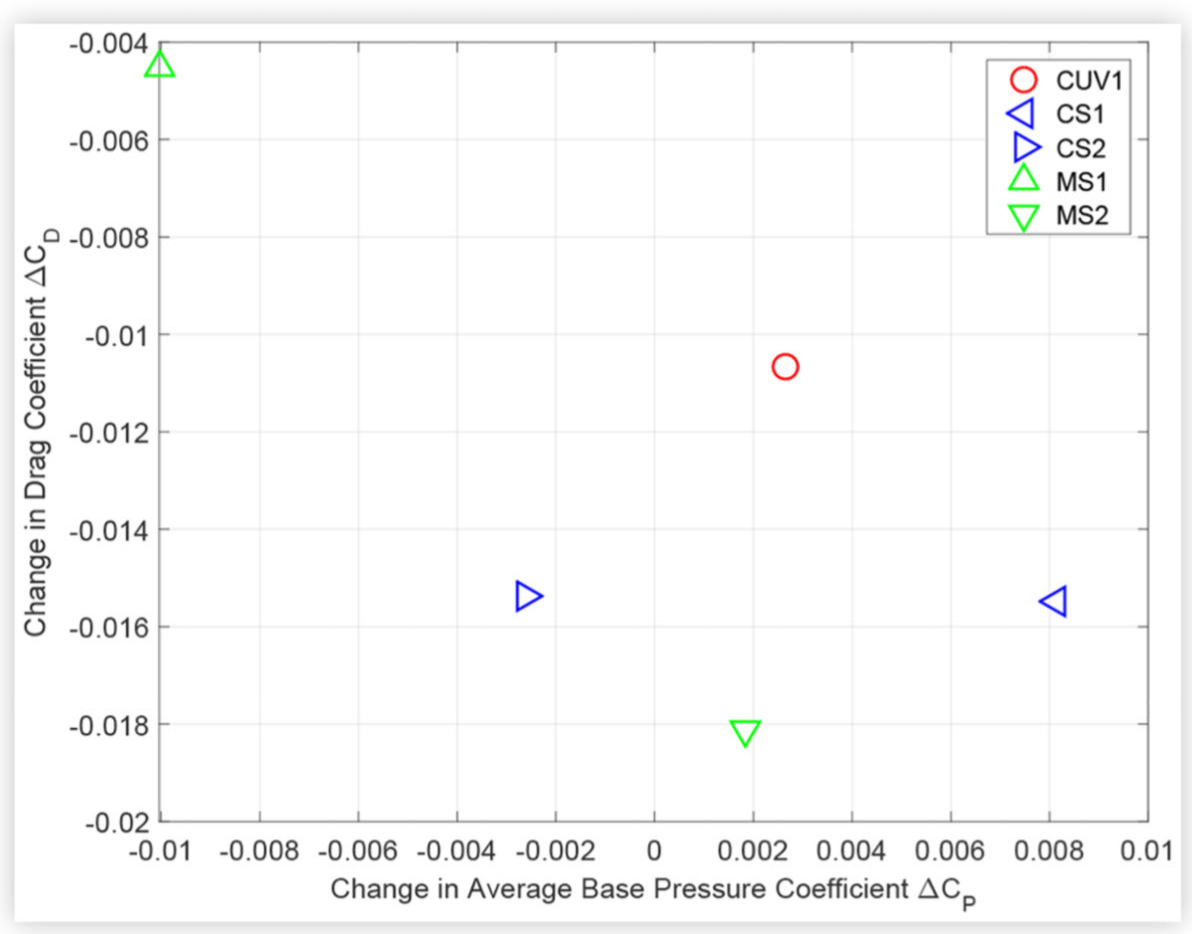
Change in drag coefficient *C*_*D*_ versus change in average base pressure coefficient *C*_*P*_ due to reducing the ride height of the test vehicles. Front and rear ride height was reduced by 30 mm for MS1, and by 40 mm for all other vehicles.

**FIGURE 15:**
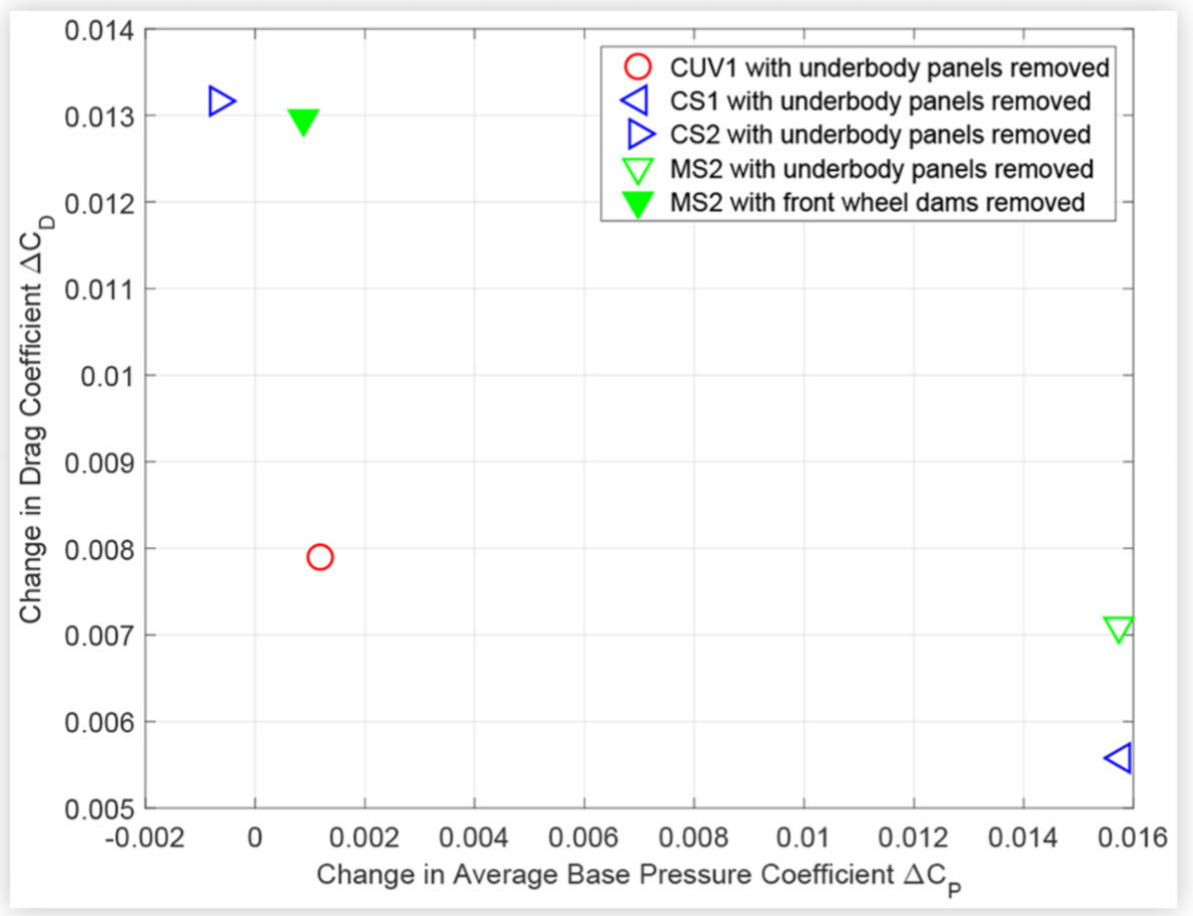
Change in drag coefficient *C*_*D*_ versus change in average base pressure coefficient *C*_*P*_ due to removing OEM underbody components (panels or wheel dams) from test vehicles.

Similarly, Figures 14 and 15 suggest an inverse relationship between changes in drag and average base pressure due to technologies such as ride height control and underbody treatments, although there is more scatter than for the cooling drag results.

Deviations from a monotonic inverse trend in the above plots could be due to the variation in the number and spatial distribution of pressure taps on the base surfaces of the vehicles. However, design variations such as grille size, AGS coverage and leakage, baseline ride height and underbody surface roughness may also have an effect. Some of these design factors are governed by engine type/size and vehicle utility segment. For example, the CUVs are equipped with internal combustion engines that require a larger grille for cooling than the electric and hybrid electric sedans, the ICE vehicles have more bluff underbody components than the BEV and HEV and the CUVs have a higher baseline ride height than the sedans. A more uniform placement and quantity of pressure taps might allow to verify if the observed trends between base pressure and drag are independent of specific vehicle design factors.

### Baseline Wake and Underbody Pressures

**FIGURE 16:**
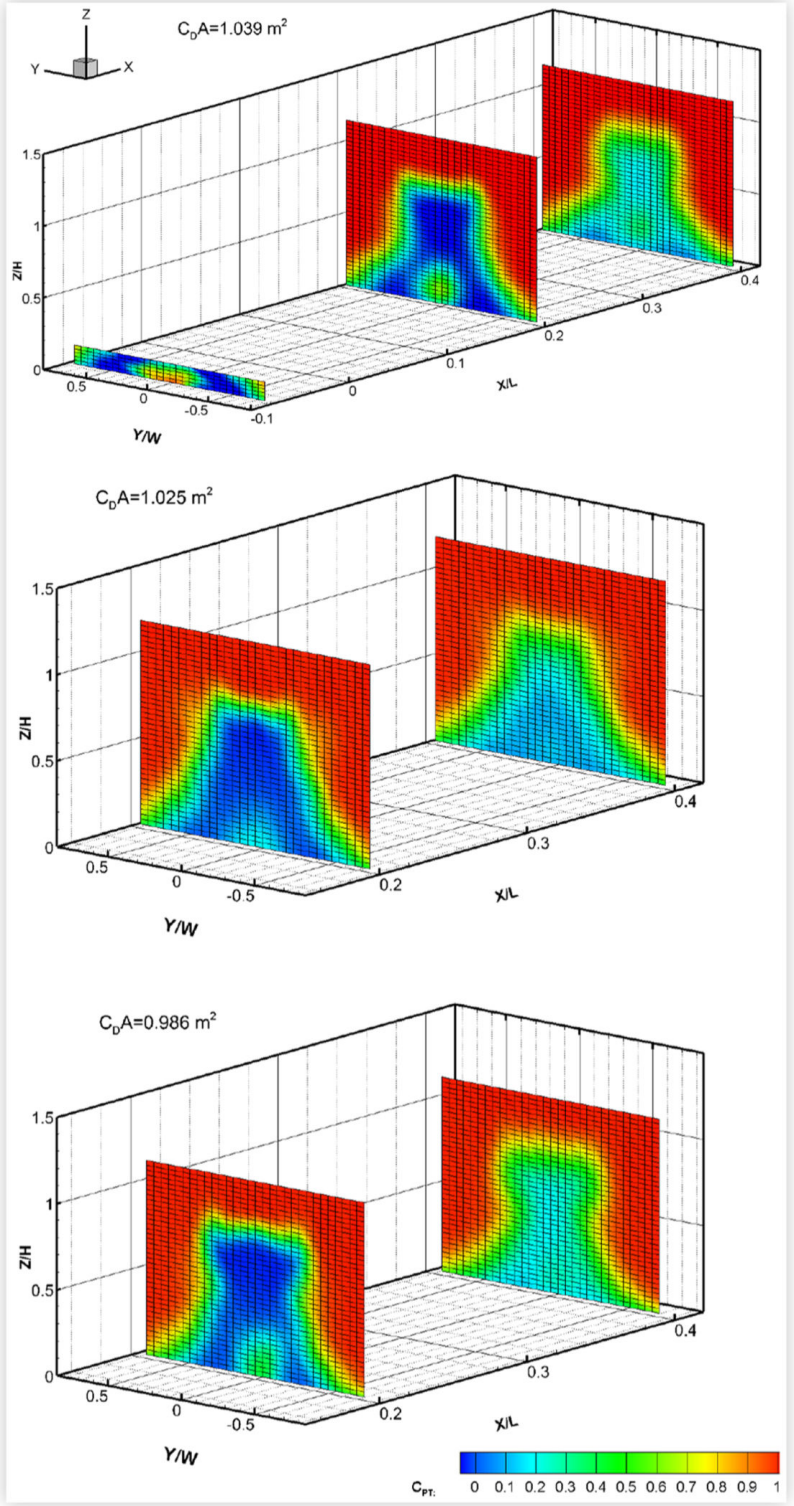
Total pressure coefficients *C*_*PT*_ in cross-flow wake/underbody planes of CUV1 at 120 km/h (top) and of CUV2 (middle) and MV1 (bottom) at 110 km/h. All vehicles were in the baseline configuration at 0° yaw in turbulent flow.

**FIGURE 17:**
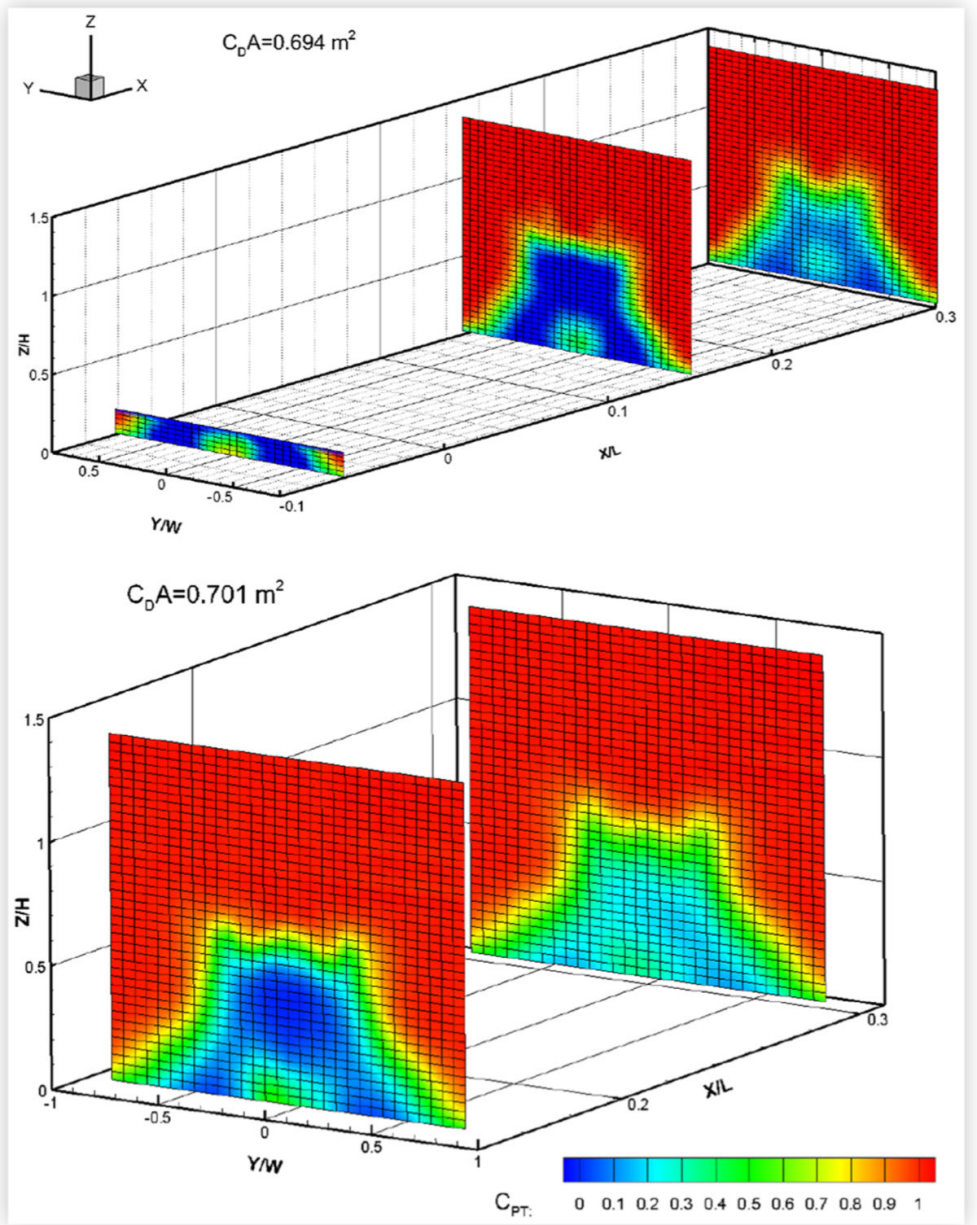
Total pressure coefficients *C*_*PT*_ in cross-flow wake/underbody planes of two compact sedans: CS1 (top) and CS2 (bottom). Both vehicles were in the baseline configuration at 0° yaw in turbulent flow at 110 km/h.

**FIGURE 18:**
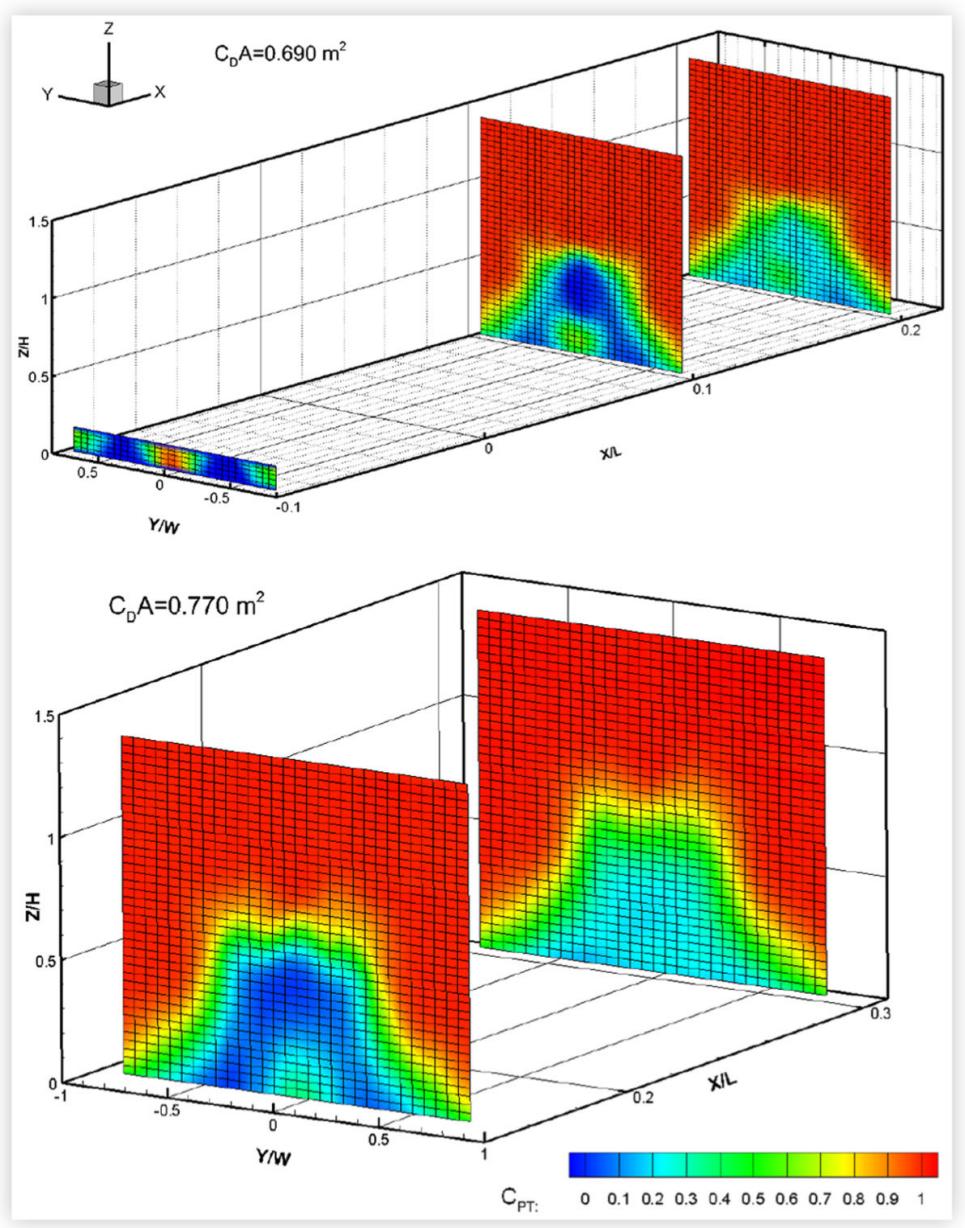
Total pressure coefficients *C*_*PT*_ in cross-flow wake/underbody planes of two mid-size sedans: MS1 (top) and MS2 (bottom). Both vehicles were in the baseline configuration at 0° yaw in turbulent flow at 110 km/h.

**FIGURE 19:**
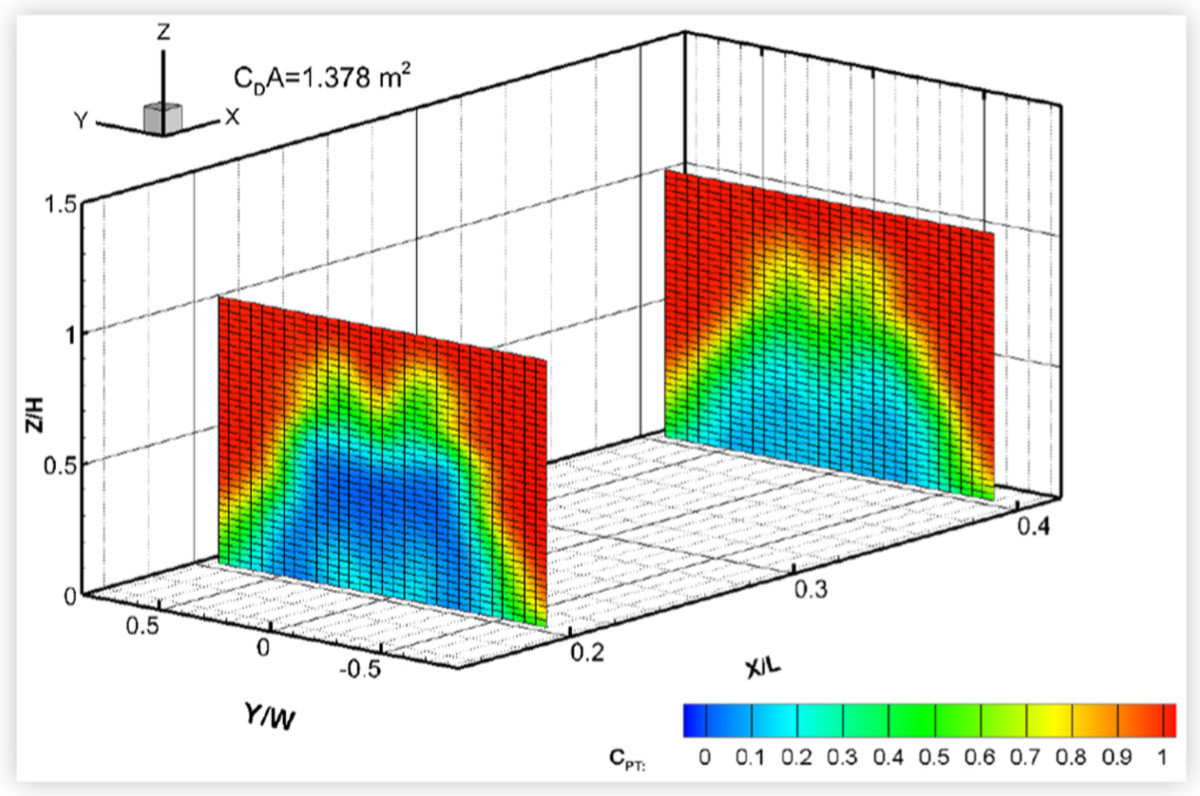
Total pressure coefficients *C*_*PT*_ in cross-flow wake planes of a pick-up truck (PU1) in the baseline configuration at 0° yaw in turbulent flow at 110 km/h.

The baseline wake total pressures measured in two cross-flow planes downstream of the eight test vehicles are shown in Figures 16 to 19. For some vehicles, total pressures were also measured in a cross-flow plane located under the vehicle between the rear wheel and the rear bumper. The exact locations of each wake/underbody plane are indicated in the figures, where *X* is measured in the streamwise direction from the most downstream point on the rear bumper, *Y* is measured from the vehicle centerline, and *Z* is measured upwards from the ground, in a right-hand coordinate system. *L*, *W* and *H* are the vehicle length, width and height, respectively. The plots show colour contours of the total pressure coefficient *C_PT_*, defined as:

(2) $$C_{PT}=\frac{\left(P_{T}-P_{S,fressiream}\right)}{Q_{frestream}}$$

where *P_T_* is the total pressure measured in the wake, and *P*_*S*, *freestream*_ and *Q_freestream_* are the blockage-corrected freestream static and dynamic pressures, respectively.

Pitot probes are known to be fairly insensitive to wind angles of up to 10° [12]. However, since there may be regions of flow in the near wake where the local wind angle at the probe tips exceeds 10°, the results will be interpreted qualitatively rather than quantitatively.

The size of the wake appeared to roughly correlate with drag area, as would be expected, but not necessarily with vehicle size. The pickup truck, with a drag area of 1.378 m^2^, had the largest wake. The wakes of the minivan and the CUVs were similar in size and smaller. Of these three vehicles, MV1 (with a drag area of 0.986 m^2^) had the narrowest wake while CUV2 had the widest, which follows the order of their drag areas. Of the four sedans, the wake and drag area of MS1 were smallest followed by those of the compact sedans. MS2 had the largest wake and drag area. All plots show the reduction in strength of the wake with streamwise distance, although all wakes remained very distinct at the most downstream wake measurement plane. The underbody pressure plots of CUV1, CS1 and MS1 clearly show the wakes of the wheels, which can still be discerned in the most downstream wake pressure plots.

## Effect of Drag Reduction Technologies on Wake Pressures

### AGS and Cooling Treatments

**FIGURE 20:**
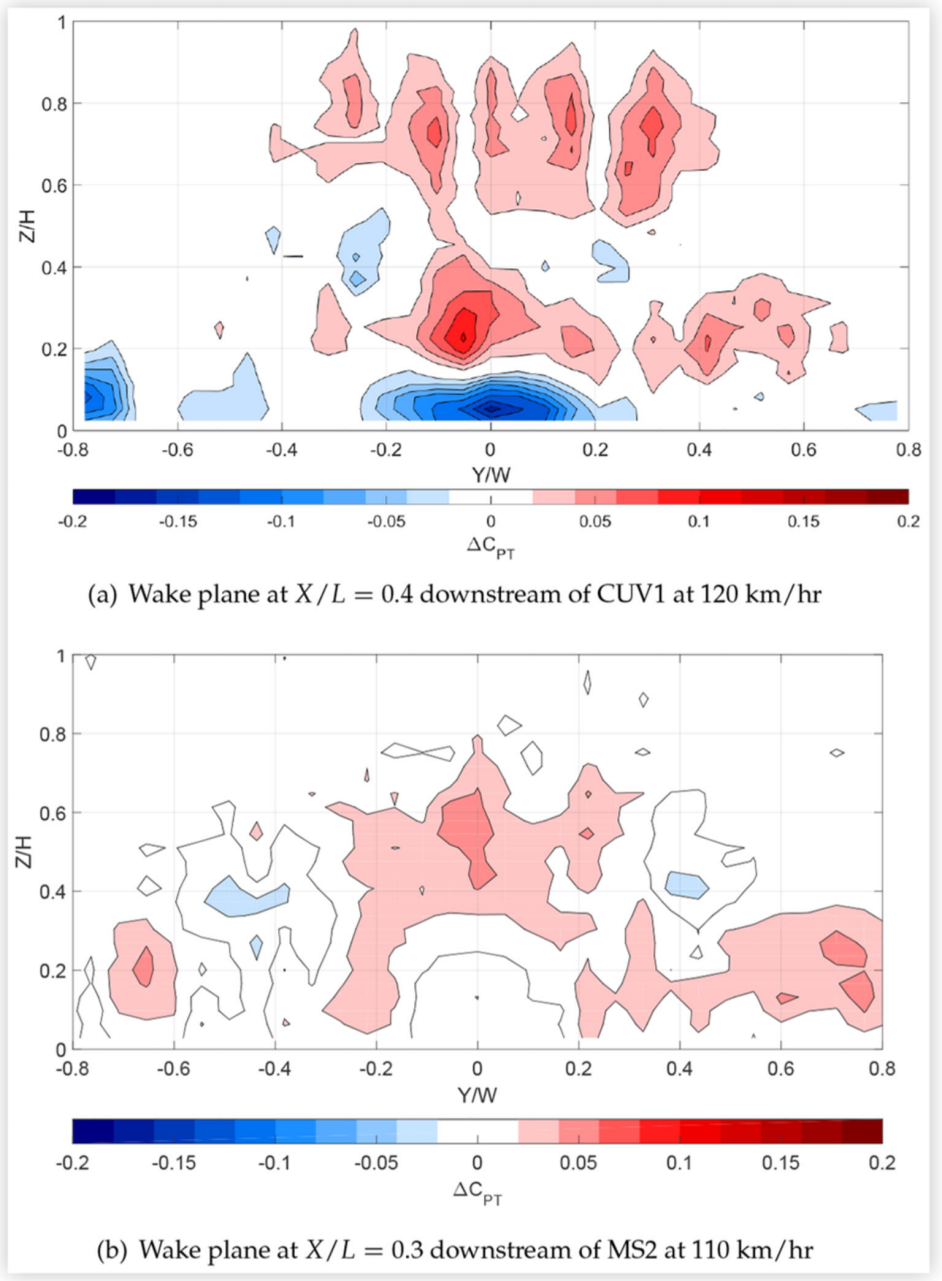
Difference in total pressure coefficients in the wake planes downstream of two vehicles at 0° yaw in turbulent flow due to closing the AGS with respect to the open shutter case: *C*_*PT*, *closed*_ − *C*_*PT*, *open*_. Viewed from downstream.

Small but measurable changes in surface pressures suggested that closing the AGS redirects air from the engine bay to above and around the vehicle. The corresponding effects on the wake pressures of CUV1 and MS2 are shown in Figure 20 and also support this hypothesis, with higher total pressures in the upper/outer part of the wake and lower total pressures near the ground. This is consistent with an increase in flow above and around the vehicle and less flow below compared to the open shutter case. It should be noted that the lower pressures in the region defined approximately by −0.2≤Y/W≤0.2 and Z/H≤0.1 in Figure 20(a) are partially due to the effects of the underbody rake track, which was only installed during tests with the shutters closed. Nevertheless, the areas of higher total pressure dominate the wake plane, which is consistent with the reduction in drag due to closing the shutters. The changes in wake pressures were stronger for CUV1, for which closing the AGS reduced drag by 2.4% compared to only 0.9% for MS2.

**FIGURE 21:**
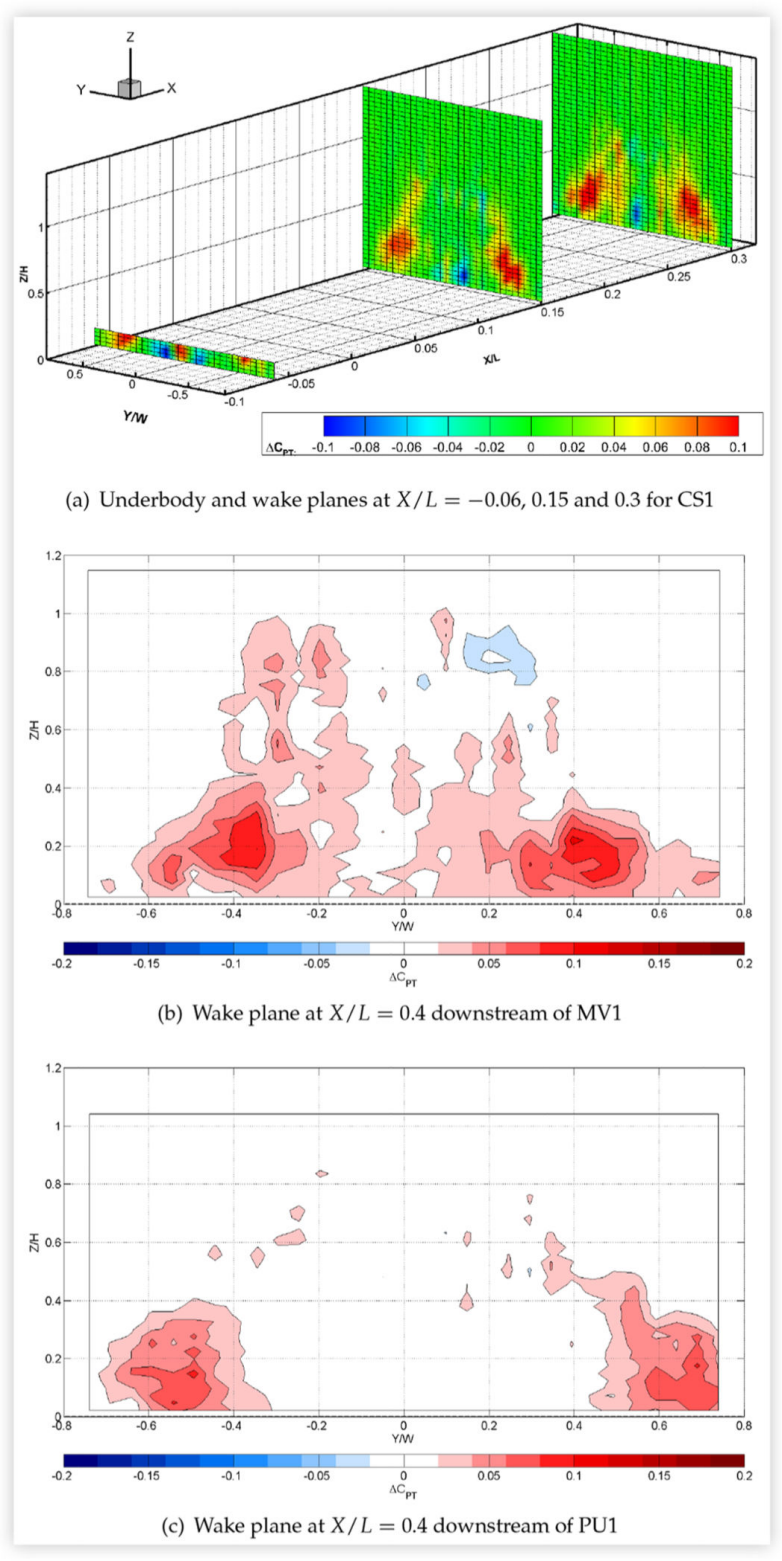
Difference in total pressure coefficients in the underbody/wake planes of three vehicles at 0° yaw in turbulent flow at 110 km/h due to sealing the external grille with respect to baseline conditions: *C*_*PT*, *sealed*_ − *C*_*PT*, *baseline*_. Viewed from downstream.

Since sealing the external grille reduced baseline vehicle drag area by up to 4.6%, it might be expected to have a commensurate effect on wake pressures. This effect was examined for seven vehicles: a CUV, all four sedans, the minivan and the pickup truck. Figure 21 shows results for the underbody and wake planes of CS1, which were typical of the sedans, and for the downstream wake planes of the minivan and pick-up truck. Pressures below baseline values were measured in small, central regions near the ground, particularly in the most upstream wake measurement plane and in the underbody plane. Pressures above baseline were measured in larger regions around the upper and/or outer periphery of the wakes of all vehicles with the grille sealed and persisted into the most downstream wake plane. These results suggest a reduction of flow beneath the vehicles and an increase in flow above and/or around the vehicles, leading to a reduction in the size of the wake.

The data presented in Figure 21 are very similar to experimental and numerical results reported by Kuthada et al. for the near wake of a quarter-scale DriveAer model [13]. Comparison of Figure 21(a) with the numerical results of these authors suggests that the regions of lower pressure in the underbody plane of CS1 on either side of its centerline axis are due to the suppression of cooling air that exits into the wheel houses and continues downstream on the inner side of the wheels, whereas the region of higher pressure at the center of the underbody plane is due to the elimination of the additional blockage caused by cooling air that exits directly under the center of the vehicle.

Regions with large increases in total pressure were particularly extensive in the wakes of both compact sedans, mid-size sedan MS2 and minivan MV1, for which reductions in drag area due to sealing the grille were highest, approaching or exceeding 4%. Measurements obtained in both wake planes of all the sedans indicated that these regions increased in size and strength moving downstream, suggesting that the wake dissipated faster with the grille sealed than at baseline conditions.

### Ride Height Control

**FIGURE 22:**
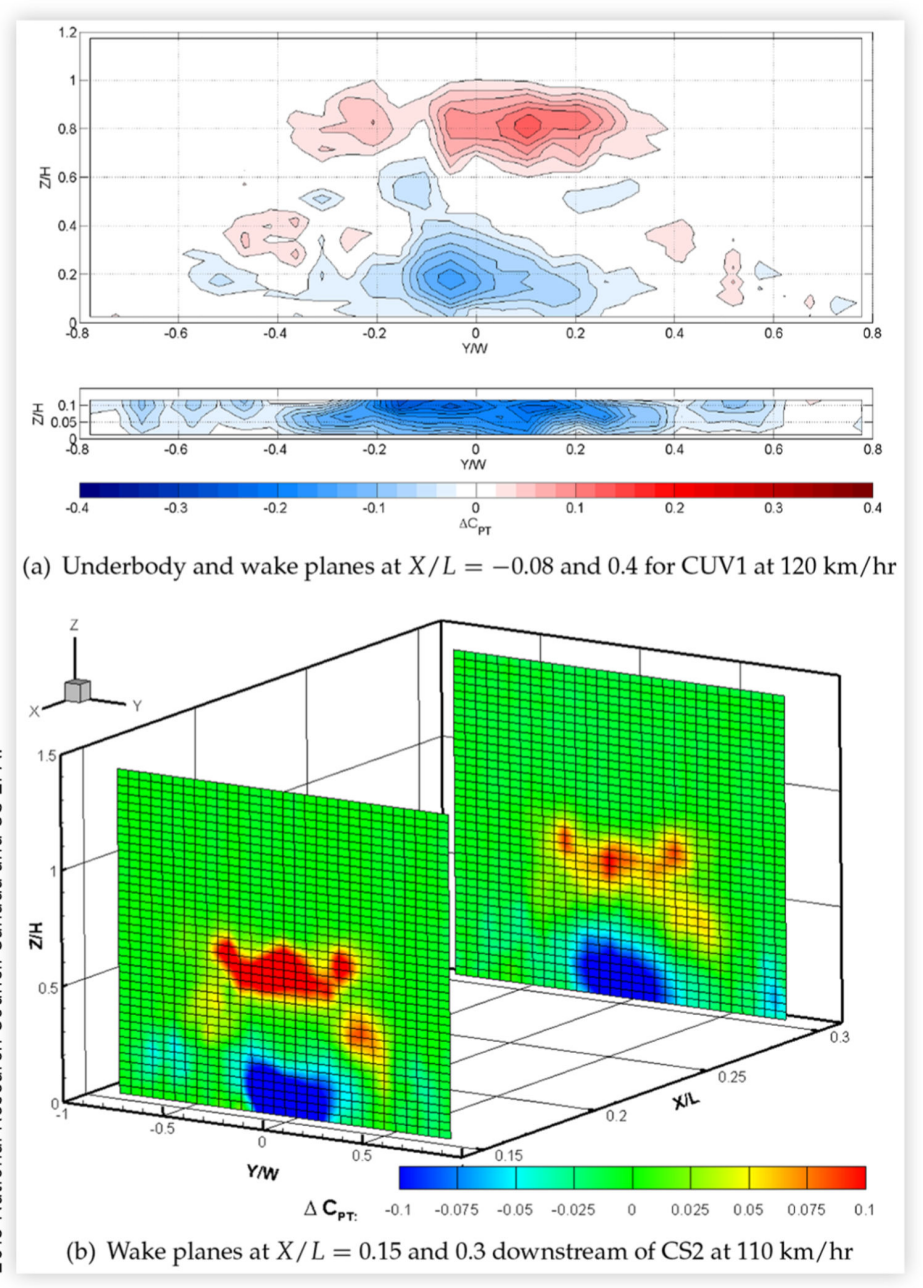
Difference in total pressure coefficients in the underbody/wake planes of two vehicles at 0° yaw in turbulent flow due to reducing the front and rear ride height by 40 mm with respect to baseline conditions: *C*_*PT*,_ − 40 _*mm*_ − *C*_*PT*, *baseline*_.

Total pressures were measured in the rear underbody and downstream wake plane of CUV1, and in both wake planes of CS2 and MS2, with the ride heights of these vehicles lowered by 40 mm. Figure 22 shows the changes in the rear underflow and/or wake pressures of CUV1 and CS2, compared to baseline values. The figure shows reduced total pressures, and thus air flow, under CUV1 and in the lower part of the wake planes, and increased pressures in the upper and, to a lesser extent, outer part of the wake planes. Similar changes were measured in both wake planes of MS2. These results, like the surface pressure measurements, are consistent with more flow being redirected from under the vehicle to above and around the vehicle due to lowering the ride height. This redistribution of flow tends to reduce the width and height of the wake, which is associated with a reduction in overall drag that adds to the reduction in wheel drag due to less exposure of the wheels to high-speed flow. The corresponding reduction in zero-yaw drag area was 3.4% for CUV1 and roughly 6% for CS2 and MS2.

The baseline wake plots of Figures 16 and 17 show that the measured wakes of CS2 and CUV1 extend to roughly 80% and 85%, respectively, of the vehicle height. Figure 22 shows that reducing the ride height by 40 mm causes a substantial increase in wake total pressure at and above 50% and 70%, respectively, of the heights of CS2 and CUV1. The vertical extent of the upper wake where total pressure is increased is over six times the ride height reduction for CUV1 and over 10 times the ride height reduction for CS2. These results suggest that lowering the ride height weakens the upper wake over a height that exceeds the ride height reduction and appears to scale with the level of drag reduction.

### Production and Custom Underbody Panels

**FIGURE 23:**
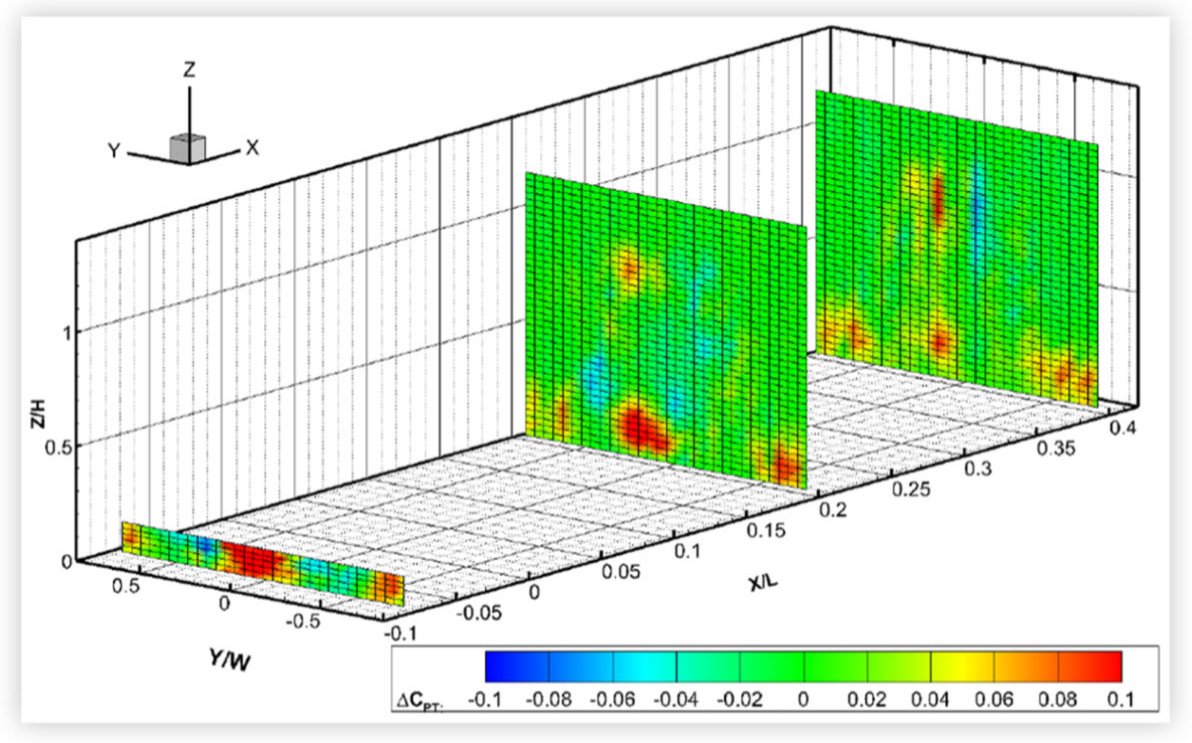
Difference in total pressure coefficients in the wake planes at *X*/*L* = 0.2 and *X*/*L* = 0.4 and in the underbody plane at *X*/*L* = − 0.08 for CUV1 at 0° yaw in turbulent flow at 120 km/h due to smoothing the underbody with custom panels with respect to baseline conditions: *C*_*PT*, *custom*_ − *C*_*PT*, *baseline*._

The custom smooth underbody panels of CUV1 reduced the zero-yaw drag area by 3.2% and had a small but measurable effect on surface pressures. The custom panels of CS1 reduced the drag area by only half as much and had a negligible effect on the measured surface pressures. Nevertheless, the custom panels caused a noticeable increase in total pressures in the central region of the underbody measurement planes and in the lower-central region of the wake planes of both vehicles. An example is shown in Figure 23 for CUV1. This is consistent with an increase in flow speed under the vehicles, and a reduction in losses due to friction drag, due to smoothing the rough underbody. For both vehicles, the region of increased total pressure in the lower-central part of the wake planes diminished in area with streamwise distance. It was noticeably smaller in the wake planes of CS1 than in those of CUV1, consistent with the lower amount of drag reduction.

**FIGURE 24:**
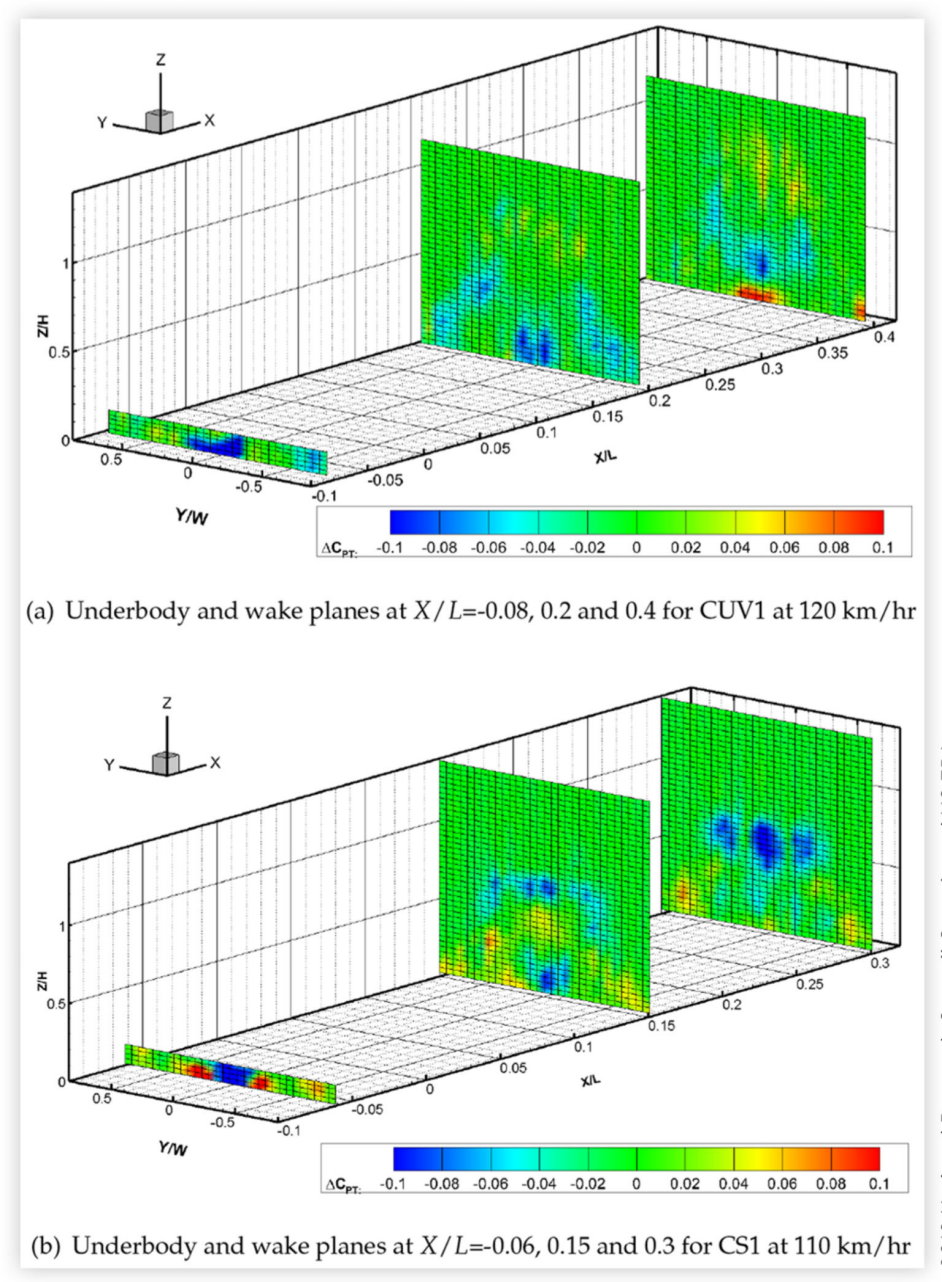
_Difference in total_ pressure coefficients in the wake and underbody planes of two vehicles at 0° yaw in turbulent flow due to removing the OEM underbody panels with respect to baseline conditions: *C*_*PT*, *no panels*_ − *C*_*PT*, *baseline*_.

Wake and underbody surveys were carried out after removing the OEM underbody panels of CUV1, both compact sedans and MS2. Only the upstream measurement planes of CS2 and MS2 were surveyed. There were significant areas of lower total pressure in the underbody and wake planes compared to baseline values, consistent with reductions in flow speed. Examples are shown for CUV1 and CS1 in Figure 24. The flow under the vehicle is slowed down as it impinges on the rough underbody surfaces, increasing overall drag. However, changes in wake pressure were not limited to the lower central portion of the wake plane and were still evident in the most downstream measurement planes of CUV1 and CS1, as shown in the figure. In particular, there were noticeable areas of reduced pressures in the upper periphery of the CS1 wake, which grew with streamwise distance, and in the outer periphery of the upstream measurement plane of CUV1 (shown in Figure 24), CS2 and MS2 (not shown). All of the results of this section suggest that smoothing the underbody of a vehicle can change the flow around the vehicle to reduce the size and strength of the wake, thereby reducing pressure drag as well as underbody friction drag.

## Summary

Surface, wake and underbody pressure measurements were found to complement force balance measurements to provide a better understanding of how aerodynamic technologies affect the flow of air around a vehicle to reduce drag. In particular, the results suggest that closing the AGS, sealing the external grille and lowering the ride height reduce drag by redirecting incoming flow from the engine bay or underbody region to smoother surfaces above and around the vehicle. This mechanism can enhance the reduction in wheel drag due to reduced wheel exposure at lowered ride height. Sealing the external grille was found to redirect the flow more efficiently than closing the grille shutters, and resulted in greater drag reduction. Underbody panels and devices reduce drag mostly by improving the underbody flow, but were found in some cases to redistribute the flow around the vehicle to reduce pressure drag as well. The magnitude and spatial extent of the measured pressure changes due to the various technologies were often consistent with the amount of drag reduction.

The wake and underbody rakes provided high spatial and pressure measurement resolution over an extensive area compared to surface pressure taps, which are usually limited in number to minimize flow disturbance. The selected surface pressure measurements were more useful to assess configuration changes that modified the air flow over a large part of the vehicle surface, such as ride height, AGS and grille sealing, than changes that had more local effects such as removing side mirrors or blocking wheel curtains, since it can be difficult to determine optimal placement of taps. On the other hand, well-positioned surface pressure measurements are efficient as they can be acquired continuously and concurrently with drag measurements whereas wake rake measurements are more time consuming due to the need for spanwise sweeps.

A correlation was found between changes in averaged base pressure measurements and changes in the vehicle drag coefficient due to various drag reduction technologies. Optimizing the number and placement of base surface pressure taps would likely improve the correlation.

## Figures and Tables

**TABLE 1:** Changes in *C*_*P*_ and *C*_*D*_*A* due to removal of side mirrors

Vehicle	Tap location(s)	Δ*CP*	Δ*CDA*
CUV1	side of rear tail light	−0.035	−2.9%
CS1	side of rear tail light	−0.016	−3.9%
CS2	none	n/a	−3.0%
MS1	side of rear tail light	+0.014	−2.7%
MS2	upper front, front of hood	−0.011, −0.017	−3.4%

## Appendix A - Test Matrix

Table 2 lists all the tests and type(s) of measurements carried out for each vehicle, where the following abbreviations are used for each measurement technique: S = surface pressure measurements, W = wake rake measurement, U = underbody rake measurements and D = drag force measurements. Blank spaces in the table indicates that the technology was either not available or not tested for the corresponding vehicle.

**TABLE 2:** Tests and type(s) of measurements carried out for each vehicle.

Vehicle Name	Baseline	AGS	Grille sealing	Ride height	Custom underbody	OEM underbody	Wheel dams	Wheel curtains	Side mirrors
CUV1	SWUD	SWUD	SWUD	SWUD	SWUD	SWUD	SD	SWD	SD
CUV2	SWD	SD		SD					
CS1	SWUD	SD	SWUD	SD	SWUD	SWUD	SD		SD
CS2	SWD		SWD	SWD		SWD		SD	SD
MS1	SWUD	SD	SWUD	SD					SWUD
MS2	SWD	SWD	SWD	SWD		SWD	SD		SD
MV1	WD	D	WD	D					
PU1	WD	D	WD	D				WD	D
